# The Epilepsy Ontology: a community-based ontology tailored for semantic interoperability and text mining

**DOI:** 10.1093/bioadv/vbad033

**Published:** 2023-03-23

**Authors:** Astghik Sargsyan, Philipp Wegner, Stephan Gebel, Abish Kaladharan, Priya Sethumadhavan, Vanessa Lage-Rupprecht, Johannes Darms, Bruce Schultz, Jürgen Klein, Marc Jacobs, Sumit Madan, Martin Hofmann-Apitius, Alpha Tom Kodamullil

**Affiliations:** Department of Bioinformatics, Fraunhofer Institute for Algorithms and Scientific Computing (SCAI), 53754 Sankt Augustin, Germany; Bonn-Aachen International Center for Information Technology (B-IT), University of Bonn, 53113 Bonn, Germany; Department of Bioinformatics, Fraunhofer Institute for Algorithms and Scientific Computing (SCAI), 53754 Sankt Augustin, Germany; Department of Bioinformatics, Fraunhofer Institute for Algorithms and Scientific Computing (SCAI), 53754 Sankt Augustin, Germany; Causality Biomodels, Kinfra Hi-Tech Park, Kalamassery, Cochin 683503, Kerala, India; Causality Biomodels, Kinfra Hi-Tech Park, Kalamassery, Cochin 683503, Kerala, India; Department of Bioinformatics, Fraunhofer Institute for Algorithms and Scientific Computing (SCAI), 53754 Sankt Augustin, Germany; German National Library of Medicine (ZB MED)—Information Centre for Life Sciences, 53115 Bonn, Germany; Department of Bioinformatics, Fraunhofer Institute for Algorithms and Scientific Computing (SCAI), 53754 Sankt Augustin, Germany; Department of Bioinformatics, Fraunhofer Institute for Algorithms and Scientific Computing (SCAI), 53754 Sankt Augustin, Germany; Department of Bioinformatics, Fraunhofer Institute for Algorithms and Scientific Computing (SCAI), 53754 Sankt Augustin, Germany; Department of Bioinformatics, Fraunhofer Institute for Algorithms and Scientific Computing (SCAI), 53754 Sankt Augustin, Germany; Department of Bioinformatics, Fraunhofer Institute for Algorithms and Scientific Computing (SCAI), 53754 Sankt Augustin, Germany; Bonn-Aachen International Center for Information Technology (B-IT), University of Bonn, 53113 Bonn, Germany; Department of Bioinformatics, Fraunhofer Institute for Algorithms and Scientific Computing (SCAI), 53754 Sankt Augustin, Germany; Causality Biomodels, Kinfra Hi-Tech Park, Kalamassery, Cochin 683503, Kerala, India

## Abstract

**Motivation:**

Epilepsy is a multifaceted complex disorder that requires a precise understanding of the classification, diagnosis, treatment and disease mechanism governing it. Although scattered resources are available on epilepsy, comprehensive and structured knowledge is missing. In contemplation to promote multidisciplinary knowledge exchange and facilitate advancement in clinical management, especially in pre-clinical research, a disease-specific ontology is necessary. The presented ontology is designed to enable better interconnection between scientific community members in the epilepsy domain.

**Results:**

The Epilepsy Ontology (EPIO) is an assembly of structured knowledge on various aspects of epilepsy, developed according to Basic Formal Ontology (BFO) and Open Biological and Biomedical Ontology (OBO) Foundry principles. Concepts and definitions are collected from the latest International League against Epilepsy (ILAE) classification, domain-specific ontologies and scientific literature. This ontology consists of 1879 classes and 28 151 axioms (2171 declaration axioms, 2219 logical axioms) from several aspects of epilepsy. This ontology is intended to be used for data management and text mining purposes.

**Availability and implementation:**

The current release of the ontology is publicly available under a Creative Commons 4.0 License and shared via http://purl.obolibrary.org/obo/epso.owl and is a community-based effort assembling various facets of the complex disease. The ontology is also deposited in BioPortal at https://bioportal.bioontology.org/ontologies/EPIO.

**Supplementary information:**

Supplementary data are available at *Bioinformatics Advances* online.

## 1 Introduction

Epilepsy is a common chronic neurological disorder, affecting more than 60 million individuals worldwide, and characterized by an enduring predisposition to generate epileptic seizures that might be associated with severe cognitive, psychological and social issues. Epileptic seizures reflect the transient occurrence of various neurological signs (motor, behavioral) and/or symptoms (cognitive, emotional, sensory), often including loss of awareness (Falco-Walter, 2020). Such seizures differ widely in severity, appearance, cause, lasting consequences and management depending on the type (Oto, 2017). Despite all available antiepileptic treatments, up to one-third of patients still suffer from uncontrolled seizures. Hence, the management of epilepsy has become a critical challenge for physicians, due in part to the difficulty in properly classifying the form of epilepsy being treated. To aid healthcare workers in this, The International League Against Epilepsy (ILAE) developed a classification system for determining the type of epilepsy affecting an individual and serves as a critical tool for practicing physicians to better understand these disease variants and their underlying mechanisms treated (Fisher *et al.*, 2014). However, in order to ensure that this classification system remains as accurate as possible, it must be updated regularly with the latest findings and reports on epilepsy. Therefore, there exists a need to create an aggregated resource of all available knowledge on epilepsy that is also compatible with the demands of clinicians, scientists, patients and industry as well as to serve as a means for accurate data collection and semantic integration. Ontologies are commonly used as semantic frameworks in biomedical and clinical knowledge disciplines for addressing these types of data management challenges. Disease-specific ontologies, such as the Cardiovascular Disease Ontology (CVDO) (Arguello Casteleiro *et al.*, 2018), incorporate disease-specific concepts and terminologies thus providing a platform for more in-depth research. The lack of a standard, specific ontology describing clinical and experimental epilepsy is a hindering detriment for both physicians and researchers. Development of a comprehensive epilepsy disease ontology that can accommodate diverse aspects, terminologies and well-defined vocabularies can serve as a core resource for epilepsy data and knowledge-driven research. In this paper, we describe an integrated Epilepsy Ontology (EPIO), which was developed in a community-based manner by various stakeholders from both Europe and the USA. The ontology was developed based on the ILAE classification of epilepsy as well as by integrating already existing epilepsy-related ontologies such as those developed according to BFO 2.0 classes and OBO Foundry principles. Many of the existing ontologies focus more on specific aspects pertaining to epilepsy, while in this work we sought to build a more generalized ontology that describes the multiple facets of epilepsy. Because the EPIO includes clinical, experimental and theoretical aspects of epilepsy, it benefits a wider range of individuals in comparison to the more focused available ontologies.

## 2 Materials and methods

Epilepsy-specific concepts were added under the upper-level classes of BFO. Protégé OWL editor (version 5.5.0) was used to build the EPIO using the ontology web language (OWL) format (https://protege.stanford.edu/). The initial version of the ontology was composed of 285 concepts with definitions from the ILAE (https://www.epilepsydiagnosis.org/). It was further enhanced using terms from other epilepsy-related ontologies such as EPSO (https://bioportal.bioontology.org/ontologies/EPSO) (Sahoo *et al.*, 2014), ESSO (https://bioportal.bioontology.org/ontologies/ESSO) and EPILONT (https://bioportal.bioontology.org/ontologies/EPILONT). Furthermore, the preclinical aspects of epilepsy include preclinical disease models, cellular and molecular disease mechanisms. Knowledge of these aspects was curated from available epilepsy-related ontologies and research articles available on PubMed. Epilepsy-associated concepts such as genes, miRNA, SNPs were not included in the ontology because of the ambiguity of the evidence associated with these. The EPIO was constructed based on guidelines and principles defined by OBO Foundry (http://www.obofoundry.org/). The Ontofox tool (http://ontofox.hegroup.org/) was used to integrate concepts from other OBO ontologies.

## 3 Results

The ontology’s structure was designed to be accessible for all users. Concepts, as well as the major classes added from the ILAE classification, were classified using the BFO 2.0 higher-order classes for proper interoperability. Multiple clinical and nonclinical sources were parsed in order to gather knowledge from epilepsy-related domains, resulting in an ontology which consists of 1879 classes and 28 151 axioms (2171 declaration axioms, 2219 logical axioms). From the clinical domain, diagnoses, clinical screening tests and treatment-related concepts were collected and added to the EPIO. Etiological, biological mechanisms, anatomical, cellular and molecular causes and effects, and preclinical disease models comprise the nonclinical epilepsy information in the ontology.

For retrieval of contextual information, we used the semantic search engine https://neuro.scaiview.com/, a repository that contains more than 5 million research articles with ∼4 million articles annotated with EPIO. The repository also contains Ontologies and Terminologies that describe other types of diseases [Alzheimer’s Disease Ontology (ADON), Human Disease Ontology (DOID), Schizophrenia Ontology (SCHIZO)], (neuro-)biological and medical expressions [Medical Subject Headings (MESH), the neuro names ontology (NN)], brain regions [the brain region cell ontology (BRCT), the FMA curated brain regions ontology (FMA)], pathways and cellular processes [the PTS pathway dictionary (PTS)], genes [HUGO Gene Nomenclature Committee (HUGO)] and compounds (DrugBank). In the analysis, terms were ordered by Kullback–Leibler divergence (REF), an information-based measure of the inequality of probability distributions.

The ontology discussed in the article acts as the semantic layer for the Text Mining-based knowledge discovery software, SCAIview (https://www.scai.fraunhofer.de/en/business-research-areas/bioinformatics/products/scaiview.html) (Malhotra *et al.*, 2015). We used a specific instance of SCAIView for neurodegenerative diseases (https://neuro.scaiview.com), which allows information retrieval with semantic searches in large text collections specific to Epilepsy. The search is carried out by means of concepts, i.e. an extended search modality through the addition of alternative terms. Concept search terms can be combined with free text search.

### 3.1 Text mining applications of Epilepsy Ontology

**Fig. 1.**
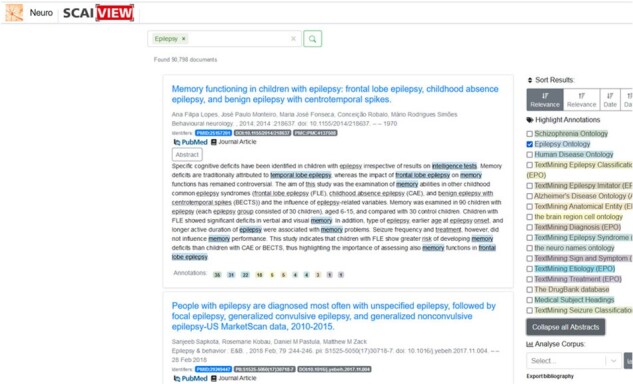
SCAIView retrieves 90 798 documents with the search term ‘*Epilepsy*’. The software automatically highlights the terms included in the Ontology

The EPIO presented here acts as the semantic layer for the Text Mining-based knowledge discovery software, SCAIview (https://www.scai.fraunhofer.de/en/business-research-areas/bioinformatics/products/scaiview.html) (Malhotra *et al.*, 2015). We used a specific instance of SCAIView for neurodegenerative diseases (https://neuro.scaiview.com), which allows information retrieval with semantic searches in large text collections specific to Epilepsy. The search is carried out by means of concepts, i.e. an extended search modality through the addition of alternative terms (Fig. 1). Concept search terms can be combined with free text search. Filtering relevant documents in the epilepsy context: using EPIO, we can filter and further process domain-specific documents. Specific filters provide information on anatomical entities, cellular processes, diagnoses, disease classifications, imitators, syndromes, etiology and risk factors in the context of epilepsy.

**Fig. 2.**
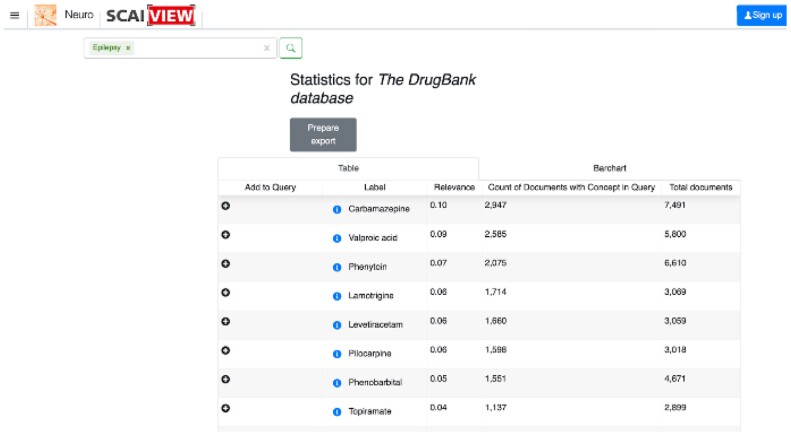
SCAIView retrieves the lists of drugs based on the relevance (how often a drug appears in the search context in relation to the total number of documents annotated with the drug)

Here, we present two examples of semantic text mining. The following biological questions were depicted:

1. Which brain regions are mainly considered in the literature in relation to brain connectivity research in epilepsy?
  In Neuro SCAIView, the EPIO was activated as a global filter and the following search parameters were entered: in the ‘I’m searching for all’ window, the concept ‘brain’ (UBERON:0000955) was used in combination with connectivity (free text). In the field ‘And in context of’ we entered the concept ‘epilepsy’ (DOID:1826). This query resulted in a corpus of 627 documents.
2. Which epilepsy-related genes overlap in different types of epilepsies? Again, the EPIO was depicted as the initial filter to search domain-specific literature. To restrict the search to documents that contain the term ‘epilepsy’ we used the corresponding concept (DOID:1826) as a search paradigm in the field ‘And in context of’. As a result, we retrieved 82 981 documents. Expanding the query with ‘temporal lobe epilepsy’ (DOID:3328) and ‘juvenile myoclonic epilepsy’ (DOID:4890) resulted in 45 documents.
  - Data mining/information extraction of metadata from epilepsy-related articles: finding the most mentioned brain region in a certain search context, then gene-specific analysis to generate a list of genes mentioned in the specific search context was applied. In addition, the EPIO, in combination with DrugBank, was used as a semantic layer to search and retrieve the epilepsy-related drugs mentioned in the literature.
    1. The resulting corpus obtained in (a) was analyzed with the text mining filter ‘Anatomical entity’. In total, the analysis yielded 54 terms, of which 47 were brain regions and 7 were nonspecific terms such as ‘brain’ or ‘axon tract’ (see Analysis Query 1 in Supplementary Information). The most mentioned brain region in our search context was ‘temporal lobe’ followed by ‘hippocampus’.
    2. The first result obtained in (b) (documents *n* = 82 981) was analyzed with the text mining filter ‘Epilepsy Classification’. After excluding the nonspecific term ‘epilepsy’, 53 descriptions of more specific epilepsy types were identified (see Analysis Query 2 Part 1 in Supplementary Information). After expanding the system query with the concepts ‘temporal lobe epilepsy’ and ‘juvenile myoclonic epilepsy’, a gene-specific analysis resulted in a list of 17 genes mentioned in the specific search context (see Analysis Query 2 Part 2 in Supplementary Information). The genes most frequently mentioned in our search context are ‘EFHC1’ (HGNC:16406), followed by ‘LGI1’ (HGNC:6572).
    3. Below is an example of how the EPIO, in combination with DrugBank, could be used as a semantic layer to search and retrieve the epilepsy-related drugs (Fig. 2). SCAIView provides the lists of drugs that are mentioned in the literature in the context of Epilepsy.
  - Grouping epilepsy articles into specific topics: the combinatorial search using epilepsy-related concepts and filters allowed for a targeted search for information and the acquisition of new knowledge based on text analytic methods that may contribute to future research. In general, epilepsy articles can be grouped into topics using the filtering options described in (a). For example, system queries in combination with relevant filters can also circumscribe a topic. Here are two situations in which this would apply:
    1. We want to narrow down a broad spectrum of articles dealing with the topic ‘Brain circuitry involvement in seizure generation’. To do this, we select a set of concepts that describe types of seizure generation, such as ‘generalized onset seizure’ (HP:0002197) and ‘focal onset seizure’ (HP:0007359) and combine this with filters that annotate domain-specific entities, cellular processes, signaling pathways, and circuits (EPIO, PTS pathway dictionary, TextMining Cellular Process).
    2. Another topic of interest is ‘neurobehavioral comorbidities of epilepsy’. To narrow down the document corpus, we choose the following parameters: We want to identify which diseases and risk factors are mentioned in the epilepsy context. For this purpose, we activate the filters ‘Epilepsy Ontology’, ‘Human Disease Ontology’ and ‘TextMining Etiology’. For detailed research on already known and possible new comorbidities in the epilepsy context, the analysis function can be used.

In summary, the application of formal knowledge representations in the form of combinatorial search using concepts and filters in the field of epilepsy allows a targeted search for information and the acquisition of new knowledge based on text analytic methods that could contribute to future research.

**Fig. 3.**
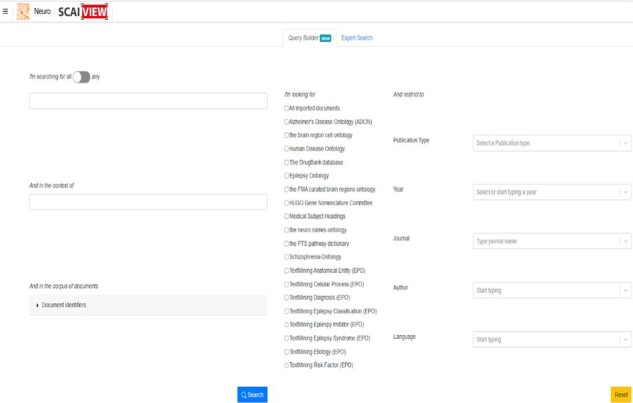
The Epilepsy BINs integrated in SCAIView which retrieves articles based on Epilepsy specific topics

SCAIView applies search query expansion to detect documents associated with a BIN (BIN referring to a epilepsy-related research topic such as ‘anatomical entity’, ‘brain connectivity’, ‘cellular process’, ‘diagnosis’, ‘epilepsy classification’, ‘epilepsy imitator’, ‘epilepsy syndrome’, ‘etiology’, ‘risk factor’, ‘seizure classification’, ‘sign and symptom’, ‘treatment’). In the EPIO ontology, we grouped the concepts within specific BINs for text mining purposes (Fig. 3).

For a given BIN identifier, the search is augmented with all subclasses of that identifier. The HermiT (http://www.hermit-reasoner.com/) reasoner is used to include asserted as well as inferred subclasses.

## 4 Discussion

We have integrated in-depth knowledge from several sources and described many crucial aspects of the disease important to the clinical and scientific communities. From the clinician’s perspective, modern intelligent systems backed up by an extensive disease-specific ontology like EPIO, may help in diagnosing and identifying the patient’s epilepsy type. The presented ontology can incorporate screening tests and the clinical history of the patient which can be utilized to gather the health record of the patient, thus serving as a useful tool by aiding clinicians in planning the treatment regimen for patients. From the researcher’s point of view, this ontology could provide all available information on associated molecular mechanisms, cell signaling pathways, preclinical models as well as the clinical details of the disease. In conclusion, the EPIO represents a substantial effort to semantically assemble the knowledge of the complex and multidimensional disease known as epilepsy. Due to the rapid growth of information and research in the field of this disease, the knowledge contained within this ontology will never be complete, and thus continuous enrichment is required. The EPIO was made publicly available so that members of the scientific community can submit open-sourced updates to it, thus helping to ensure the ontology stays as current as possible.

## Supplementary Material

vbad033_Supplementary_Data
