# Supplementary material for: The Epilepsy Ontology: a community-based ontology tailored for semantic interoperability and text mining
Source: Bioinform Adv. 2023 Mar 23;3(1):vbad033. doi: 10.1093/bioadv/vbad033 (PMC10067149; doi:10.1093/bioadv/vbad033)
Supplement: vbad033_Supplementary_Data [file vbad033_supplementary_data.zip › EpilepsyOnt- Supplementary file_FINAL.pdf]

Supplementary Document

Building of the Epilepsy ontology

Supplementary text/table 1: Collection of Concepts

In order to build the content of the EPIO Ontology, we collected concepts from different sources, including already existing Epilepsy related ontologies (ESSO, Epilepsy and Seizure Ontology, EPILONT), the International League Against Epilepsy (ILAE) website, as well as other domain specific resources and publications. Detailed resource information is described below.

- Initially, 285 concepts and definitions provided by the International League Against Epilepsy (ILAE) website (https://www.epilepsydiagnosis.org/) were collected and integrated in the EPIO Ontology.
- Epilepsy related ontologies have been selected and checked. Afterwards, the included concepts have been inspected and new terms were added.
    - Additionally, initial comparison between ontologies was performed before the integration of terms. The results are summarised in the table below:

Name	Scope	Total no. of Concepts	No. of Common Concepts	No. of Uncommon Concepts	Reference
EPIO	The Epilepsy Ontology (EPIO) is an assembly of structured knowledge on various aspects of epilepsy, developed according to basic formal ontology (BFO) and Open Biological and Biomedical Ontology (OBO) Foundry principles. Entities and definitions are collected from the latest International League against Epilepsy (ILAE) classification, as well as from domain-specific ontologies such as Epilepsy Ontology (EPILONT), Epilepsy Syndrome Seizure Ontology	1916	-	-	• http://purl.obolibrary.org/obo/epio.owl<br>• ttps://bioportal.bioontology.org/ontologies/EPIO

	(ESSO), Epilepsy Semiology(EPISEM) and Epilepsy and Seizure Ontology (EPSO) and scientific literature.				
ESSO	This ontology contains epilepsy syndromes, seizure types, and data elements associated with them.	2705	356	2349	https://bioportal.bioontology.org/ontologies/ESSO
Epilepsy and Seizure Ontology	The Epilepsy and Seizure Ontology (EpSO) is an application ontology developed to support epilepsy focused informatics tools for patient care and clinical research.	1357	171	1186	https://bioportal.bioontology.org/ontologies/EPSO
EPiLONT	Ontology about the epilepsy domain and epileptic seizures. Based on the diagnosis proposed by the International League Against Epilepsy (ILAE).	138	36	102	https://bioportal.bioontology.org/ontologies/EPILONT

Supplementary Table 3: Comparison to other related ontologies (as of November 13, 2020)

Note: The comparison on concepts was done automatically. This is not very accurate as many of the above mentioned ontologies have not imported concepts from previously existing ontologies or have not re-used the concepts. Therefore, an exact comparison of concept to concept was not feasible.

- Relevant entities and concepts were also collected from 459 research articles. The list of PMIDs of the used articles are mentioned below.

Number	PMID/DOI	Title
1	30233290	In vitro Models for Seizure-Liability Testing Using Induced Pluripotent Stem Cells
2	12181003	Heart rate changes and ECG abnormalities during epileptic seizures: prevalence and definition of an objective clinical sign
3	29110774	Epilepsy
4	2384077	Head circumference in children of epileptic mothers: contributions of drug exposure and genetic background
5	25179745	Obesity and its association with generalised epilepsy, idiopathic syndrome, and family history of epilepsy
6	26575850	Cerebrospinal fluid findings after epileptic seizures
7	28288363	The role of postictal laboratory blood analyses in the diagnosis and prognosis of seizures
8	23538271	Coexistence of epilepsy and Brugada syndrome in a family with SCN5A mutation
9	14594442	Seizures in alcohol-dependent patients: epidemiology, pathophysiology and management
10	24441294	Tobacco smoking, epilepsy, and seizures
11	27025991	[Episodic ataxia type 2 manifests as epileptiform electroencephalographic activity with no epileptic attacks in two family members]

12	31327513	Frequency of takotsubo cardiomyopathy in epilepsy-related hospitalizations among adults and its impact on in-hospital outcomes: A national standpoint
13	30691036	Epilepsy in Children with Autistic Spectrum Disorder
14	28775613	SEIZURE RELATED SEXUAL DISORDER
15	29237192	Pediatric Klüver-Bucy Syndrome: Report of Two Cases and Review of the Literature
16	12503648	Benign familial infantile seizures: further delineation of the syndrome
17	15797356	Educational problems with underlying neuropsychological impairment are common in children with Benign Epilepsy of Childhood with Centrotemporal Spikes (BECTS)
18	22916156	Epilepsy is a risk factor for sudden cardiac arrest in the general population
19	20430655	A patient with DiGeorge syndrome with spina bifida and sacral myelomeningocele, who developed both hypocalcemia-induced seizure and epilepsy
20	1831121	Allergic rash due to antiepileptic drugs: clinical features and management
21	26198661	Two Patients With Visual Aura - Migraine, Epilepsy, or Migralepsy?
22	28671982	Clinical services for adults with an intellectual disability and epilepsy: A comparison of management alternatives
23	30562654	Seizures as a clinical manifestation in somatic autoimmune disorders
24	29213881	Epilepsy in patients with Alzheimer's disease: A systematic review
25	11864518	[Renal failure as a factor leading to epileptic

		seizures]
26	31055731	Hypertension, seizures, and epilepsy: a review on pathophysiology and management
27	11104349	Association of epilepsy with different groups of microcephaly
28	2912820	Differentiating multiple personality disorder and complex partial seizures
29	28749241	Considerations for ADHD in the child with epilepsy and the child with migraine
30	28043687	Hemiconvulsion-Hemiplegia-Epilepsy Syndrome
31	31437864	Depersonalization- and derealization-like phenomena of epileptic origin
32	28360564	Self-Esteem, Social Phobia and Depression Status in Patients with Epilepsy
33	3579680	Transient global amnesia and epilepsy. Electroencephalographic distinction
34	20161538	Association of epilepsy and comorbid conditions
35	30279715	The Relation between Urinary Tract Infection and Febrile Seizure
36	32034533	Epilepsy in brain metastasis: an emerging entity
37	27531853	[Repeated non-epileptic seizures in a previously healthy young woman - a case report]
38	25387857	Standardized assessment of seizures in patients with juvenile neuronal ceroid lipofuscinosis
39	27629553	Neuronal ceroid lipofuscinoses
40	26423537	Infections, inflammation and epilepsy
41	32149989	Treatment Outcomes of Pediatric Status Epilepticus in a Tertiary Pediatric Intensive Care Unit

42	18777476	Recognition of nonepileptic events
43	9532709	[Epilepsy in patients with ischemic brain disease]
44	21741275	Epilepsy surgery in children with developmental tumours
45	15347872	Spectrum of congenital CNS malformations in pediatric epilepsy
46	26478444	Seizures in oligodendroglial tumors
47	31552392	Zolpidem dependence in an adult with bipolar affective disorder and epilepsy: A case report
48	18472483	Mood disorder and epilepsy: a neurobiologic perspective of their relationship
49	32014726	Long-term follow-up of post-stroke epilepsy after ischemic stroke: Room for improved epilepsy treatment
50	30022372	Serum Uric Acid Is Highly Associated with Epilepsy Secondary to Cerebral Infarction
51	31604333	Preoperative tumor-associated epilepsy in patients with supratentorial meningioma: factors influencing seizure outcome after meningioma surgery
52	21851492	A rare cause of cardiac syncope mimicking epilepsy: left main coronary artery stenosis
53	26132164	Dysgraphia as a Mild Expression of Dystonia in Children with Absence Epilepsy
54	6527775	The role of brain edema in epileptic brain damage induced by systemic kainic acid injection
55	31361236	Pigmented variant of pleomorphic xanthoastrocytoma - A rare long-term epilepsy associated neoplasm
56	26362394	Thyroid hormones: Possible roles in epilepsy

		pathology
57	30924504	Predictors of Epileptic Seizures and Ability to Work in Supratentorial Cavernous Angioma Located Within Eloquent Brain Areas
58	8252282	Epilepsy and schizophrenia
59	19655091	Myocardial infarction due to late stent thrombosis following epileptic convulsive seizures
60	30865078	Depressive Symptom Severity in Individuals With Epilepsy and Recent Health Complications
61	10474720	HIV infection and seizures
62	27856781	Parental rheumatoid arthritis and childhood epilepsy: A nationwide cohort study
63	20301555	Aicardi Syndrome
64	26549780	Comorbidities of epilepsy: current concepts and future perspectives
65	29379967	Development of Neuromyelitis Optica Spectrum Disorder and Spinal Arachnoid Cysts in a Patient With Intractable Epilepsy
66	24687183	Population-level evidence for an autoimmune etiology of epilepsy
67	24073547	Epilepsy in children with subacute sclerosing panencephalitis
68	25214788	Risk of epilepsy in patients with systemic lupus erythematosus - a retrospective cohort study
69	29791879	Seizure imitators monitored using video-EEG in children with intellectual disabilities
70	29241678	Language Dysfunction in Pediatric Epilepsy
71	29720810	Recovery of Visual Scotomas by Vortioxetine in a Patient with Symptomatic Occipital Lobe Epilepsy

72	30600130	Epilepsy in children with type 1 diabetes mellitus: Pathophysiological basis and clinical hallmarks
73	26468872	Delayed Intraparenchymal Hematoma Following Diagnostic Lumbar Puncture
74	30661434	From next-generation sequencing to targeted treatment of non-acquired epilepsies
75	27430454	Neuroimaging of epilepsy
76	27789166	How neuropsychology can improve the care of individual patients with epilepsy. Looking back and into the future
77	21490734	Role of single photon emission computed tomography in epilepsy
78	29722352	Epilepsy
79	14537108	Positron emission tomography and epilepsy
80	22554135	The effect of seizure focus on regional language processing areas
81	17855377	Stereoelectroencephalography in presurgical assessment of MRI-negative epilepsy
82	26721354	Epileptogenic effects of G protein-coupled estrogen receptor 1 in the rat pentylenetetrazole kindling model of epilepsy
83	28089585	The impact of intelligence on memory and executive functions of children with temporal lobe epilepsy: Methodological concerns with clinical relevance
84	18976727	A homozygous mutation in human PRICKLE1 causes an autosomal-recessive progressive myoclonus epilepsy-ataxia syndrome
85	2782045	Eating epilepsy
86	17371290	Apoptosis signalling pathways in seizure-induced neuronal death and epilepsy

87	29961525	Focal Epilepsy in a Teenager With Facial Atrophy and Hair Loss
88	12365699	Unusual eye movements in a patient with complex partial seizure disorder
89	8441366	Behavioral changes associated with epilepsy
90	28139515	Clinical Analysis of Partial Epilepsy with Auras
91	20301709	Autosomal Dominant Epilepsy with Auditory Features
92	17260039	Depression and epilepsy: a new perspective on two closely related disorders
93	13365719	A case of precocious puberty with epileptic seizure
94	30838190	Precocious and Early Central Puberty in Children With Pre-existing Medical Conditions: A Single Center Study
95	29172092	Visual field defects after temporal lobe resection for epilepsy
96	31603835	[Epilepsy in children with congenital hemiparesis secondary to perinatal ictus]
97	27188686	Cerebral palsy
98	2761703	Seizures after primary intracerebral hemorrhage
99	26864574	When the face says it all: dysmorphology in identifying syndromic causes of epilepsy
100	30118929	Ictal nausea and vomiting - Is it left or right?
101	30125861	The role of magnetoencephalography in the presurgical evaluation of patients with MRI-negative operculo-insular epilepsy
102	12199726	Interictal EEG abnormalities in patients with psychogenic nonepileptic seizures
103	29656099	Hypsarrhythmia in epileptic spasms: Synchrony

		in chaos
104	30378543	Sleep-related hypermotor epilepsy and peri-ictal hypotension in a patient with syntaxin-1B mutation
105	30666028	Valproic acid-induced nocturnal enuresis in pediatric patients
106	15642493	Symptoms in focal sensory seizures. Clinical and electroencephalographic features
107	27857611	Epilepsy and the Sensory Systems
108	10919145	[Anatomic substrate of epigastric aura: case report]
109	15911361	Somatosensory auras in focal epilepsy: a clinical, video EEG and MRI study
110	26249726	The relevance of somatosensory auras in refractory temporal lobe epilepsies
111	12134331	[Epileptic auras: classification, pathophysiology, practical usefulness, differential diagnosis and controversials]
112	12609279	Integration of Perceptual and Mnemonic Dysfunction: Sensory Auras Are Associated with Left Hemispheric Memory Impairment
113	30068811	[A case of painful seizure accompanying ictal paresis and homonymous hemianopia due to post-stroke epilepsy]
114	22791548	Provider practices impact adequate diagnosis of sleep disorders in children with epilepsy
115	30661063	Brain Morphology in Patients with Genetic Generalized Epilepsy: Its Heterogeneity among Subsyndromes
116	28488762	Visual Auras in Epilepsy and Migraine - An Analysis of Clinical Characteristics
117	23365482	Vasovagal syncope treated as epilepsy for 16

		years
118	20627778	Partial epilepsy presenting as focal atonic seizure: a case report
119	6820630	[Tonic epileptic crisis and complex and mixed atypical absences in Lennox-Gastaut syndrome in patients over 6 years of age]
120	22580903	Obstructive sleep apnea and primary snoring in children with epilepsy
121	22776676	Complex epileptic palilalia: a case report
122	26088882	Effect of epileptic seizures on the cerebrospinal fluid--A systematic retrospective analysis
123	22844307	Focal cortical dysplasia - review
124	31085954	Pathological Classification of Focal Cortical Dysplasia (FCD) : Personal Comments for Well Understanding FCD Classification
125	30711777	Parasites and epilepsy: Understanding the determinants of epileptogenesis
126	19744116	Nonconvulsive status epilepticus and coma
127	26336950	A definition and classification of status epilepticus--Report of the ILAE Task Force on Classification of Status Epilepticus
128	26920416	Treatment of Convulsive Status Epilepticus
129	24977129	Recurrent seizures following focal motor status epilepticus in a patient with non-ketotic hyperglycemia and acute cerebral infarction
130	7469854	Acute epidural hematoma following epileptic seizures
131	25905906	Cognitive impairment in epilepsy: the role of network abnormalities
132	25856437	Revealing Medicinal Plants That Are Useful for the Comprehensive Management of Epilepsy and Associated Comorbidities through In Silico

		Mining of Their Phytochemical Diversity
133	30473971	Postictal Mania Versus Postictal Psychosis
134	9579937	Dissociation in epilepsy and conversion nonepileptic seizures
135	16650147	Epileptic seizures superimposed on catatonic stupor
136	9291731	Formal thought disorder and psychopathology in pediatric primary generalized and complex partial epilepsy
137	26167207	Assessing the prevalence distribution of abnormal laboratory tests in patients with simple febrile seizure
138	31911412	Transient seizure-induced sodium increase camouflaging a symptomatic hyponatremia
139	23534590	[Cerebral creatine deficiency syndromes]
140	25777785	Major congenital malformations in children of women with epilepsy
141	11076003	Venous angiomas and epilepsy
142	24134485	Cavernoma-related epilepsy: review and recommendations for management--report of the Surgical Task Force of the ILAE Commission on Therapeutic Strategies
143	31578307	Activity of hippocampal adult-born neurons regulates alcohol withdrawal seizures
144	30782577	A novel mutation in KCNQ3-related benign familial neonatal epilepsy: electroclinical features and neurodevelopmental outcome
145	2512371	Epileptic seizures in intracerebral haemorrhage
146	15146004	Clinical features and long term outcome of epilepsy in periventricular nodular heterotopia. Simple compared with plus forms
147	28658095	A clinical review on megalencephaly: A large

		brain as a possible sign of cerebral impairment
148	31830676	New-onset refractory status epilepticus: A retrospective cohort study
149	32062735	Novel mutation of SCN9A gene causing generalized epilepsy with febrile seizures plus in a Chinese family
150	28671587	Inborn Errors of Metabolism and Epilepsy: Current Understanding, Diagnosis, and Treatment Approaches
151	26592968	Postencephalitic epilepsy and drug-resistant epilepsy after infectious and antibody-associated encephalitis in childhood: Clinical and etiologic risk factors
152	6955943	Attentional and perceptual disturbances in children with Tourette's syndrome, attention deficit disorder, and epilepsy
153	28116304	Alice in Wonderland Syndrome: A Clinical and Pathophysiological Review
154	8500435	Epilepsy in the setting of neurocutaneous syndromes
155	24761136	Post-traumatic epilepsy: an overview
156	23551133	Complications of epilepsy surgery: a systematic review of focal surgical resections and invasive EEG monitoring
157	32038177	The Epilepsy of Infancy With Migrating Focal Seizures: Identification of de novo Mutations of the KCNT2 Gene That Exert Inhibitory Effects on the Corresponding Heteromeric K Na 1.1/K Na 1.2 Potassium Channel
158	25917466	Febrile seizures and genetic epilepsy with febrile seizures plus (GEFS+)
159	21135885	The role of inflammation in epilepsy
160	17984449	Obstructive sleep apnea is associated with

		seizure occurrence in older adults with epilepsy
161	26441491	Modulation of autonomic activity in neurological conditions: Epilepsy and Tourette Syndrome
162	20696621	Hormonal alterations following seizures
163	25966854	Asystole in the epilepsy unit
164	26394714	Genotype-phenotype correlation of congenital anomalies in multiple congenital anomalies hypotonia seizures syndrome (MCAHS1)/PIGN-related epilepsy
165	27768938	Hyperammonaemia and associated factors in unprovoked convulsive seizures: A cross-sectional study
166	25180909	Causes and consequences of gray matter heterotopia
167	7487261	Value of tongue biting in the diagnosis of seizures
168	29414563	Genital automatisms: Reappraisal of a remarkable but ignored symptom of focal seizures
169	16103019	The role of serotonin in impulsive and aggressive behaviors associated with epilepsy-like neuronal hyperexcitability in the amygdala
170	29287215	Impact of vagus nerve stimulation on sleep-related breathing disorders in adults with epilepsy
171	10996566	NREM parasomnias: arousal disorders and differentiation from nocturnal frontal lobe epilepsy
172	19162231	Focal epileptic seizures mimicking sleep paralysis
173	32086099	Focal cortical hypermetabolism in atypical benign rolandic epilepsy

174	21633606	Management of provoked seizure
175	31617494	Intermittent photic stimulation-provoked seizure associated with ictal asystole
176	29325826	Myoclonic absence seizures with complex gestural automatisms
177	31832260	High-dose Clozapine Withdrawal: A Case Report and Timeline of a Single Potential Withdrawal Seizure
178	15528919	A note on gelastic epilepsy
179	29170920	Dacrystic Epilepsy
180	22938964	Animal models
181	28332054	A Mesiotemporal Lobe Epilepsy Mouse Model
182	29933054	Imaging correlates of behavioral impairments: An experimental PET study in the rat pilocarpine epilepsy model
183	23929939	Modeling human neurodevelopmental disorders in the Xenopus tadpole: from mechanisms to therapeutic targets
184	13268119	Epilepsy in Macaca mulatta after cortical or intracerebral alumina
185	30219655	Circadian and circaseptan rhythms in human epilepsy: a retrospective cohort study
186	23190285	Common imitators of epilepsy
187	24679945	The utility score of epilepsy with partial seizure measured by TTO, VAS, and EQ-5D in the general Korean population
188	22512895	A comparison of quality of life in adolescents with epilepsy or asthma using the Short-Form Health Survey (SF-36)
189	11081816	Daytime sleepiness in epilepsy patients: evaluation by means of the Epworth sleepiness scale

190	28064112	Ultra-short screening instruments for major depressive episode and generalized anxiety disorder in epilepsy: The NDDIE-2 and the GAD-SI
191	30599368	Hospital Anxiety and Depression Scale-Anxiety subscale (HADS-A) and The State-Trait Anxiety Inventory (STAI) accuracy for anxiety disorders detection in drug-resistant mesial temporal lobe epilepsy patients
192	31026785	Reliability and validity of the Chinese version of the Patient Health Questionnaire 9 (C-PHQ-9) in patients with epilepsy
193	26921599	Associations of impaired sleep quality, insomnia, and sleepiness with epilepsy: A questionnaire-based case-control study
194	21576040	Self-reported medication adherence and treatment satisfaction in patients with epilepsy
195	26255305	Diffusion MRI and its Role in Neuropsychology
196	27914224	A systematic review of epileptic seizures in adults with subdural haematomas
197	25204010	Intraoperative ElectroCorticoGraphy (ECog): indications, techniques, and utility in epilepsy surgery
198	31914330	The adverse-effect profile of lacosamide
199	26874864	Interictal epileptiform discharge effects on neuropsychological assessment and epilepsy surgical planning
200	23941480	Occupational therapy and epilepsy
201	31461681	Usefulness of preschool and school versions of the Behavioral Rating Inventory of Executive Functions in the evaluation of the daily life executive function in myoclonic-atonic epilepsy
202	22258041	Montreal Cognitive Assessment in cryptogenic epilepsy patients with normal Mini-Mental

		State Examination scores
203	29991426	Positive Airway Pressure Therapy Is Challenging for Patients With Epilepsy
204	21849000	Effect of continuous positive airway pressure treatment on seizure control in patients with obstructive sleep apnea and epilepsy
205	27101469	The role of MEG in pre-surgical evaluation of epilepsy: current use and future directions
206	30819542	Autonomic biofeedback therapy in epilepsy
207	20345933	Alterations of intracerebral γ-aminobutyric acid (GABA) levels by titration with levetiracetam in patients with focal epilepsies
208	24861650	The preoperative evaluation and surgical treatment of epilepsy
209	29140112	A 2017 review of pharmacotherapy for treating focal epilepsy: where are we now and how will treatment develop?
210	27091679	Descriptive study of symptomatic epilepsy by age of onset in patients with a 3-year follow-up at the Neuropaediatric Department of a reference centre
211	30909075	Default mode network deactivation in pediatric temporal lobe epilepsy: Relationship to a working memory task and executive function tests
212	28324301	Neuroimaging in Epilepsy
213	15281961	Magnetoencephalography in epilepsy
214	8215194	Computed tomography in patients with recurrent seizures
215	23250841	Critical review of palliative surgical techniques for intractable epilepsy
216	21269290	Epilepsy and the new cytogenetics

217	30086482	In vitro and in vivo experimental models employed in the discovery and development of antiepileptic drugs for pharmacoresistant epilepsy
218	20492865	[Experimental models in epilepsy]
219	30030085	Deep brain stimulation for epilepsy
220	28384785	Maternal Body Mass Index in Early Pregnancy and Risk of Epilepsy in Offspring
221	27210239	Psychiatric and Behavioural Disorders in Children with Epilepsy: an ILAE Task Force Report
222	16380232	Epilepsy, surgery, and the elderly
223	29111504	Validation of the Polish version of the Beck Depression Inventory in patients with epilepsy
224	29624147	Risk factors for surgical site infection after intracranial electroencephalography monitoring for epilepsy in the pediatric population
225	29382476	[Factors associated with epilepsy in children in Mexico: A case-control study]
226	19243383	Global expression profiling in epileptogenesis: does it add to the confusion?
227	28382495	Cognitive and Behavioral Interventions in Epilepsy
228	30591281	Laser interstitial thermotherapy (LiTT) in pediatric epilepsy surgery
229	21909104	Glutamate release by primary brain tumors induces epileptic activity
230	25819950	Classical neurotransmitters and neuropeptides involved in generalized epilepsy in a multi-neurotransmitter system: How to improve the antiepileptic effect?

231	25487080	Antiepileptic drugs influences on body weight in people with epilepsy
232	28858722	Comprehension and production of nouns and verbs in temporal lobe epilepsy
233	26293325	When patients with epilepsy or "epilepsy" might need a pacemaker
234	22693291	Progressive seizures in a patient with congenital coagulopathies
235	25260205	Idiopathic brain herniation. A report of two paediatric cases
236	29888583	[A Meta-analysis of the Effectiveness of Acupuncture in the Treatment of Epilepsy]
237	26033084	Seizures and epilepsy: an overview for neuroscientists
238	28190753	Evaluation of the first seizure patient: Key points in the history and physical examination
239	29107258	Epilepsy: Clinical Review and Surgical Options
240	28951736	Classification of Hand Grasp Kinetics and Types Using Movement-Related Cortical Potentials and EEG Rhythms
241	23515147	Dietary therapies for epilepsy
242	15608956	Intelligence functions disorders in patients with complex partial epilepsy
243	2871721	Kindling model of epilepsy
244	16059497	Sleep deprivation and epilepsy
245	3064627	Theta rhythms in the EEG: a genetic trait in childhood epilepsy
246	10908505	Yoga for epilepsy
247	13789853	Traumatic epilepsy after closed head injuries
248	14630489	Use of aromatherapy (with or without

		hypnosis) in the treatment of intractable epilepsy--a two-year follow-up study
249	26835338	Hemispherectomy in the treatment of seizures: a review
250	30760973	Ketogenic Diet and Epilepsy: What We Know So Far
251	22826811	Vagus nerve stimulation for epilepsy: A review of the peripheral mechanisms
252	28214547	Cerebral small vessel disease predisposes to temporal lobe epilepsy in spontaneously hypertensive rats
253	24686330	Slow modulations of high-frequency activity (40-140-Hz) discriminate preictal changes in human focal epilepsy
254	28532712	Photosensitivity and epilepsy: Current concepts and perspectives-A narrative review
255	32154929	Peri-ictal responsiveness to the social environment is greater in psychogenic nonepileptic than epileptic seizures
256	26677173	Inhibition of p38 mitogen-activated protein kinase signaling reduces multidrug transporter activity and anti-epileptic drug resistance in refractory epileptic rats
257	27346214	Immune response in the eye following epileptic seizures
258	30912766	Pro-inflammatory, IL-17 pathways dominate the architecture of the immunome in pediatric refractory epilepsy
259	29467619	Cannabidiol Regulates Long Term Potentiation Following Status Epilepticus: Mediation by Calcium Stores and Serotonin
260	30767241	New insights into human lysine degradation pathways with relevance to pyridoxine-dependent epilepsy due to antiquitin deficiency

261	23252947	Association of carbamazepine major metabolism and transport pathway gene polymorphisms and pharmacokinetics in patients with epilepsy
262	24114605	Atypical vitamin B6 deficiency: a rare cause of unexplained neonatal and infantile epilepsies
263	29359340	Review: Mechanistic target of rapamycin (mTOR) pathway, focal cortical dysplasia and epilepsy
264	29737480	Notch Signaling Regulates Microglial Activation and Inflammatory Reactions in a Rat Model of Temporal Lobe Epilepsy
265	26260962	Epilepsy and innate immune system: A possible immunogenic predisposition and related therapeutic implications
266	24914213	Protein degradation and quality control in cells from laforin and malin knockout mice
267	29512697	Ephrin- b3 modulates hippocampal neurogenesis and the reelin signaling pathway in a pilocarpine- induced model of epilepsy
268	30262417	Brain-derived neurotrophic factor and epilepsy: a systematic review
269	17910583	Alterations of phosphatidylinositol 3-kinase pathway components in epilepsy-associated glioneuronal lesions
270	25221392	Pharmacogenetic evaluation of ABCB1, Cyp2C9, Cyp2C19 and methylene tetrahydrofolate reductase polymorphisms in teratogenicity of anti-epileptic drugs in women with epilepsy
271	25433904	Altered glutamate protein co-expression network topology linked to spine loss in the auditory cortex of schizophrenia
272	28074534	Depolarizing γ-aminobutyric acid contributes to glutamatergic network rewiring in epilepsy

273	24723228	Calcium signaling and epilepsy
274	26254980	Genetically epilepsy-prone rats (GEPRs) and DBA/2 mice: Two animal models of audiogenic reflex epilepsy for the evaluation of new generation AEDs
275	25312505	Advances on genetic rat models of epilepsy
276	28506440	The Wistar Audiogenic Rat (WAR) strain and its contributions to epileptology and related comorbidities: History and perspectives
277	11219629	Hydrocephalus and epilepsy
278	25535236	Skin lesions in a patient with epilepsy
279	30416962	Recurrent focal seizures as a feature of status epilepticus presenting as a peri-ictal water drinking
280	22698381	Prolonged atrial fibrillation following generalized tonic-clonic seizures
281	22637287	Atrial fibrillation associated with epileptic seizures
282	18539570	Unilateral mydriasis during temporal lobe seizures
283	30698542	[Anxiety disorders in epilepsy]
284	23142708	The diagnostic value of urinary incontinence in the differential diagnosis of seizures
285	27638925	Mechanisms of memory impairment in epilepsy depend on age at disease onset
286	29763181	Simple Partial Seizure
287	18754955	Bacterial meningitis and epilepsy
288	15562299	Effects of Seizures on Autonomic and Cardiovascular Function
289	23613463	Low-frequency electrical stimulation of a fiber tract in temporal lobe epilepsy

290	23671345	Subcortical epilepsy?
291	23622192	Brain maturation and epilepsy
292	5096551	Focal dysplasia of the cerebral cortex in epilepsy
293	3987648	Effect of midbrain and pontine tegmental lesions on audiogenic seizures in genetically epilepsy-prone rats
294	23810707	Midbrain-hindbrain malformations in patients with malformations of cortical development and epilepsy: a series of 220 patients
295	26368332	Olfactory Bulbectomy Leads to the Development of Epilepsy in Mice
296	22091816	Ventricular enlargement in new-onset pediatric epilepsies
297	28912749	Functional Connectivity of the Corpus Callosum in Epilepsy Patients with Secondarily Generalized Seizures
298	31920906	Superior Frontal Sulcus Focal Cortical Dysplasia Type II: An MRI, PET, and Quantified SEEG Study
299	26063964	The Classical Pathways of Occipital Lobe Epileptic Propagation Revised in the Light of White Matter Dissection
300	15857433	Entorhinal cortex involvement in human mesial temporal lobe epilepsy: an electrophysiologic and volumetric study
301	30269938	Distinctive epileptogenic networks for parietal operculum seizures
302	21403025	Cingulate gyrus epilepsy: clinical and behavioral aspects, with surgical outcomes
303	30100562	Cingulate gyrus epilepsy
304	28928072	Orbitofrontal epilepsy: Case series and review

		of literature
305	21925841	EEG background activity is abnormal in the temporal and inferior parietal cortex in benign rolandic epilepsy of childhood: a LORETA study
306	26295917	A case of secondary somatosensory epilepsy with a left deep parietal opercular lesion: successful tumor resection using a transsubcentral gyral approach during awake surgery
307	10775518	Quantitative MRI volumetry of the entorhinal cortex in temporal lobe epilepsy
308	29519472	Parietal lobe epilepsy
309	11377839	Evidence of peripheral auditory activity modulation by the auditory cortex in humans
310	18209276	Depression, hopelessness and suicide risk among patients suffering from epilepsy
311	29960852	Improving compliance in adults with epilepsy on a modified Atkins diet: A randomized trial
312	11684349	Expression of vesl-1S/homer-1a, a gene associated with long-term potentiation, in the brain of the epileptic EI mouse
313	1834893	A slow intravenous infusion of N-methyl-DL-aspartate as a seizure model in the mouse
314	25568300	The phenotypic spectrum of SCN8A encephalopathy
315	31044310	Comparing the Wada Test and Functional MRI for the Presurgical Evaluation of Memory in Temporal Lobe Epilepsy
316	25228809	Animal models of epilepsy: use and limitations
317	25845493	Cross-species pharmacological characterization of the allylglycine seizure model in mice and larval zebrafish

318	21532379	Frontal lobe seizures
319	9932952	Anterior temporal abnormality in temporal lobe epilepsy: a quantitative MRI and histopathologic study
320	30714986	Autoimmune seizures and epilepsy
321	30139784	Ictal cardiorespiratory depression: a real risk for sudden unexpected death in epilepsy (SUDEP)?
322	28324947	A sparse Laguerre-Volterra autoregressive model for seizure prediction in temporal lobe epilepsy
323	2345619	Characterization of the basal temporal language area in patients with left temporal lobe epilepsy
324	20231918	Benign EEG patterns: is there more to learn?
325	11292215	Benign myoclonus of early infancy: an imitator of West's syndrome
326	15032388	Benign neonatal sleep myoclonus mimicking status epilepticus
327	16302879	Nonepileptic disorders imitating generalized idiopathic epilepsies
328	1396544	Chemical models of epilepsy with some reference to their applicability in the development of anticonvulsants
329	29623857	The Large Conductance Calcium- and Voltage-activated Potassium Channel (BK) and Epilepsy
330	1283140	Effects of antiepileptic drugs, calcium channel blockers and other compounds on seizures induced by activation of voltage-dependent L calcium channel in DBA/2 mice
331	15941650	Benign childhood epilepsy with occipital paroxysms: neuropsychological findings

332	9024190	Functional anatomy of spontaneous seizures in a rat model of limbic epilepsy
333	10802766	Anterior cingulate gyrus epilepsy: the role of ictal rCBF SPECT in seizure localization
334	30528098	Conventional and quantitative EEG in status epilepticus
335	25769270	Animal models of temporal lobe epilepsy following systemic chemoconvulsant administration
336	10510977	Corneal kindling in mice: behavioral and pharmacological differences to conventional kindling
337	22995680	Computational models of epilepsy
338	23027094	Dorsolateral frontal lobe epilepsy
339	25687591	EEG guidelines in the diagnosis of brain death
340	24405074	Stereotactic placement of depth electrodes in medically intractable epilepsy
341	27093945	Extrastriate visual cortex in idiopathic occipital epilepsies: The contribution of retinotopic areas to spike generation
342	7925156	Extratemporal cortical resections and lesionectomies for partial epilepsy: complications of surgical treatment
343	17162190	Ictal EEG in benign partial epilepsy in infancy
344	30057567	Clinical Management of Epilepsy With Glutamic Acid Decarboxylase Antibody Positivity: The Interplay Between Immunotherapy and Anti-epileptic Drugs
345	20868357	In vivo experimental models of epilepsy
346	17525024	Focal motor seizure with automatisms in a newborn
347	16563807	Cognitive problems related to epilepsy

		syndromes, especially malignant epilepsies
348	23667393	Dissociative seizures: a challenge for neurologists and psychotherapists
349	24649451	Simple Partial Status of Forced Thinking Originated in the Mesial Temporal Region: Intracranial Foramen Ovale Electrode Recording and Ictal PET
350	22832396	Cognitive dysfunction with complex visual hallucinations due to focal nonconvulsive status epilepticus: a neuropsychological study and SISCOM
351	29376090	Cognitive impairment in epilepsy: the role of reduced network flexibility
352	19846832	Directed aggressive behavior in frontal lobe epilepsy: a video-EEG and ictal spect case study
353	26924970	Ecstatic Epileptic Seizures: A Glimpse into the Multiple Roles of the Insula
354	30361137	Movement disorders phenomenology in focal motor seizures
355	8848969	Epileptic negative myoclonus
356	19679190	Evidence that juvenile myoclonic epilepsy is a disorder of frontotemporal corticothalamic networks
357	11554899	Gait epilepsy. A case report of gait-induced seizures
358	29327337	Generalized nonmotor (absence) seizures-What do absence, generalized, and nonmotor mean?
359	19423297	Delusions, illusions and hallucinations in epilepsy: 1. Elementary phenomena
360	8021672	Heautoscopy, epilepsy, and suicide
361	24777136	Epilepsy and the hippocampus
362	27070861	Effects of hippocampal partial kindling on

		sensory and sensorimotor gating and methamphetamine-induced locomotion in kindling-prone and kindling-resistant rats
363	21030341	Hypnopompic seizures
364	26038597	Cardiac arrhythmias during or after epileptic seizures
365	26304794	Ictal pattern on scalp EEG at onset of seizure in temporal lobe epilepsy: Old and new problems for epileptologists
366	15329073	Clinical manifestations of insular lobe seizures: a stereo-electroencephalographic study
367	28637636	Epilepsy after cerebral infection: review of the literature and the potential for surgery
368	12744361	Complications of invasive subdural grid monitoring in children with epilepsy
369	6149233	The jittery newborn and infant: a review
370	19286474	Gelastic seizures: A case of lateral frontal lobe epilepsy and review of the literature
371	15788545	Occipital epilepsy: lateral versus mesial
372	23872083	Hypermotor seizures in lateral and mesial parietal epilepsy
373	26317672	Neurocognitive Changes Associated With Surgical Resection of Left and Right Temporal Lobe Glioma
374	21896690	Mesial temporal sclerosis in epilepsy
375	31846897	Low CSF CD4/CD8+ T-cell proportions are associated with blood-CSF barrier dysfunction in limbic encephalitis
376	25667835	Ictal kissing with subdural EEG recording
377	1324090	Ontogenic study of lithium-pilocarpine-induced status epilepticus in rats

378	23027093	Mesial frontal lobe epilepsy
379	23027091	Mesial temporal lobe epilepsy
380	6133913	Alterations in the content of amino acid neurotransmitters before the onset and during the course of methoxypyridoxine-induced seizures in individual rabbit brain regions
381	24632481	Study of psychiatric comorbidities in epilepsy by using the Mini International Neuropsychiatric Interview
382	20627816	The diagnostic utility of the ictal cry
383	1915173	Benign partial epilepsy of childhood with monomorphic sharp waves in centrotemporal and other locations
384	9757434	Multilobar resections in surgical treatment of medically intractable epilepsy
385	8194189	Multiple sleep latency tests in epilepsy
386	12908748	Negative and positive visual hypnotic hallucinations: attending inside and out
387	23531441	Head atonic attacks: a new type of benign non-epileptic attack in infancy strongly mimicking epilepsy
388	2369876	Differentiation of epileptic from nonepileptic head drops in children
389	9109891	Noninvasive assessment of language dominance in children and adolescents with functional MRI: a preliminary study
390	9832214	Non-invasive assessment of language dominance with near-infrared spectroscopic mapping
391	7631075	Role of multichannel magnetoencephalography in the evaluation of ablative seizure surgery candidates

392	27627857	Non-invasive Evaluation for Epilepsy Surgery
393	24163755	NON-INVASIVE BRAIN STIMULATION IN CHILDREN: APPLICATIONS AND FUTURE DIRECTIONS
394	12684556	Clinical correlation of occipital intermittent rhythmic delta activity
395	15489401	Visual disturbances representing occipital lobe epilepsy in patients with cerebral calcifications and coeliac disease: a case series
396	23027097	Occipital lobe seizures and epilepsies
397	9128446	Occipital pole area and language dominance
398	835966	Ocular oscillations
399	2909707	Oligoantigenic diet treatment of children with epilepsy and migraine
400	24881594	Prevention of organophosphate-induced chronic epilepsy by early benzodiazepine treatment
401	23384238	The use of organotypic slice cultures for the study of epileptogenesis
402	24592228	Out-of-body experiences associated with seizures
403	19453720	Parahippocampal epilepsy with subtle dysplasia: A cause of "imaging negative" partial epilepsy
404	30909077	The neuropsychological profile of parietal and occipital lobe epilepsy
405	2779593	The sz mutant hamster: a genetic model of epilepsy or of paroxysmal dystonia?
406	29151098	The Alice-in-Wonderland Syndrome
407	18762233	Penicillin-induced epilepsy model in rats: dose-dependant effect on hippocampal volume and neuron number

408	21939841	Electrical stimulation for epilepsy: experimental approaches
409	8330586	West syndrome due to perinatal insults
410	29804730	Perinatal insults and neurodevelopmental disorders may impact Huntington's disease age of diagnosis
411	19333408	Visual hallucinations: differential diagnosis and treatment
412	24251565	Models in research of pharmacoresistant epilepsy: present and future in development of antiepileptic drugs
413	20618423	In search of models of pharmacoresistant epilepsy
414	2924747	Phenytoin-induced seizures: a paradoxical effect at toxic concentrations in epileptic patients
415	11956004	Picrotoxin-induced generalised convulsive seizure in rat: changes in regional distribution and frequency of the power of electroencephalogram rhythms
416	11762207	Animal models of schizophrenia: a critical review
417	18550176	The pilocarpine model of temporal lobe epilepsy
418	16954451	Post-stroke seizure and post-stroke epilepsy
419	23926279	Posterior cingulate epilepsy: clinical and neurophysiological analysis
420	8154869	Posterior temporal epilepsy: electroclinical features
421	10908200	Role of primary sensorimotor cortices in generating inhibitory motor response in humans

422	10924866	Comparison between the QOLIE-31 and derived QOLIE-10 in a clinical trial of levetiracetam
423	12639061	Responsiveness of the quality of life in epilepsy inventory (QOLIE-89) in an antiepileptic drug trial
424	30215021	Responsive neurostimulation for epilepsy: More than stimulation
425	12427900	Singing seizures
426	23959883	Two distinct forms of functional lateralization in the human brain
427	25012363	How can we identify ictal and interictal abnormal activity?
428	22957229	Selective amygdalohippocampectomy
429	24861272	Routine developmental, autism, behavioral, and psychological screening in epilepsy care settings
430	26365965	A Special Electroencephalography Pattern Might Help in the Diagnosis of Antibody-positive Encephalitis
431	6537832	Speech arrest and supplementary motor area seizures
432	32144451	[Epilepsy in old age]
433	29755904	Radiation-induced Cavernous Malformation as a Late Sequelae of Stereotactic Radiosurgery for Epilepsy
434	2804800	Temporal intermittent rhythmic delta activity (TIRDA) in the diagnosis of complex partial epilepsy: sensitivity, specificity and predictive value
435	21704564	Benign temporo-parieto-occipital junction epilepsy with vestibular disturbance: an underrecognized form of epilepsy?

436	12615636	Occipital epilepsies: identification of specific and newly recognized syndromes
437	15632275	The out-of-body experience: disturbed self-processing at the temporo-parietal junction
438	26002462	Absence-like seizures and their pharmacological profile in tottering-6j mice
439	26307329	Neuromodulation in the Treatment of Epilepsy
440	23002376	1.5 tesla magnetic resonance imaging scanners compared with 3.0 tesla magnetic resonance imaging scanners: systematic review of clinical effectiveness
441	15572277	Adenosine A1 receptors and the anticonvulsant potential of drugs effective in the model of 3-nitropropionic acid-induced seizures in mice
442	20955719	The 4-aminopyridine in vitro epilepsy model analyzed with a perforated multi-electrode array
443	24013377	Experimental models of status epilepticus and neuronal injury for evaluation of therapeutic interventions
444	20951004	Comparative anticonvulsant efficacy in the corneal kindled mouse model of partial epilepsy: Correlation with other seizure and epilepsy models
445	2395534	The C57BL/10Bg sps/sps mouse: a mutant with absence-like seizures; neurochemical and behavioral correlates
446	31961886	Health-related quality of life and its determinants among ambulatory patients with epilepsy at Ambo General Hospital, Ethiopia: Using WHOQOL-BREF
447	23862049	Palpitations caused by a Seizure with Autonomic Features
448	25988019	Ictal bradycardia and atrioventricular block: a

		cardiac manifestation of epilepsy
449	22050551	Seizure-induced miosis
450	16060951	Seizures manifesting as an urge to defecate, with an ictal discharge in the right hemisphere
451	10932282	Ictal urinary urge indicates seizure onset in the nondominant temporal lobe
452	29686574	Urinary urge seizure semiology localization by intracranial monitoring
453	22957231	Déjà experiences in temporal lobe epilepsy
454	21478188	Neural correlates of cognitive impairment in posterior cortical atrophy
455	14667072	Acquired epileptic dysgraphia: a longitudinal study
456	11039971	Partial seizures presenting as panic attacks
457	15562304	Distinguishing Lateral Temporal Neocortical and Mesial Temporal Lobe Epilepsy
458	24967532	Ontogenic profile of seizures evoked by the beta-carboline DMCM (methyl-6,7-dimethoxy-4-ethyl-β-carboline-3-carboxylate) in rats
459	27657542	The Natural History of Epilepsy in 163 Untreated Patients: Looking for "Oligoepilepsy"

Supplementary Table 1: Research articles and reviews considered to extract the main concepts

- In addition, we used the following websites to enhance the ontology with more significant concepts:

**Website name**	**URL**
Stanford Health Care	https://stanfordhealthcare.org/medical-conditions/brain-and-nerves/epilepsy.html

American Association of Neurological Surgeons	https://www.aans.org/Patients/Neurosurgical-Conditions-and-Treatments/Epilepsy

Supplementary Table 2: Websites considered to collect concepts

Supplementary text 2: Construction of the ontology

The EPIO Ontology was assembled using the Protégé ontology editor. This ontology is constructed based on guidelines and principles defined by Open Biological and Biomedical Ontology (OBO, http://www.obofoundry.org/) Foundry as well as aligned with the Basic Formal Ontology (BFO) hierarchy. Wherever possible, the hierarchy was based on the parent ontology from which the concept was imported. We applied Ontofox (http://ontofox.hegroup.org) to reuse previously existing classes from other relevant ontologies. For each concept, most of the OBO ontology sources were identified, and the concepts were imported with all available annotations via Ontofox. Undefined terms in other ontologies were added with proper definitions as well as with clear provenance. In order to increase recall in Text Mining applications, we added synonyms for each concept.

Supplementary text 3: Metadata information using annotation properties

We added a large amount of metadata information using various annotation properties. Annotation properties were added to each entity in the ontology. These properties explain the name of the entity, standard ontology from which the entity is imported, definition of the term, source from which the term is adopted, and references that connect the term to Epilepsy. The details of the annotation properties are listed below:

- **label**: Label annotation is the unique display name of the concept.
- **oboInowl:hasDefinition**: Definitions for the concept are added under this annotation property
- **rdfs:isDefinedBy**: This annotation is added as an internal annotation to definitions, to point to the source of the definition when the definition is added manually. For modified definitions, the source of the definition is given under oboInOwl:hasDbXRef or rdfs:seeAlso.
- **oboInOwl:hasDbXRef**: To add an epilepsy specific reference link from Pubmed/NCBI
- **rdfs:seeAlso**: Any additional relevant reference links such as web articles are given under rdfs:seeAlso.
- **oboInOwl:hasExactSynonym**: Synonyms are added under this annotation property. Synonyms are terms derived from articles or research papers
- **oboInOwl:hasRelatedSynonym**: The terms that are related synonyms of the entity are added under this annotation property.

Moreover, in regards to the aforementioned annotation properties, we added some custom annotation properties to define additional information as well as to aid in applying the ontology for the use cases defined in the manuscript. Some custom annotations were used to note the source from which the terms are taken, specifying if the definition is modified from the source article of the definition and giving an explanation of the BFO hierarchy of the term. These custom annotations are listed below:

- **fromPubMed** - concepts are taken from PubMed article
- **fromArticle** - concepts are taken from resources other than PubMed
- **fromNCBIBook** - concepts are taken from NCBI Books
- **fromILAE -** concepts are taken from International League Against Epilepsy
- **fromESSO** - concept are taken from the existing ontology ESSO (Epilepsy Syndrome Seizure Ontology)
- **fromEpSO -** concept are taken from the existing ontology EpSO (Epilepsy and Seizure Ontology)
- **fromEPILONT -** concept are taken from the existing ontology EPILONT (Epilepsy Ontology)
- **CommentonDefinition** - to mention that given definition taken from the source is modified.

In addition, here is the list of annotations added to the concepts, which are used further to apply in text-mining use cases:

- **isAnatomicalEntityFor**: for concepts assigned to the "anatomical entity TM_BIN" BIN
- **isCellularProcessFor**: for concepts assigned to the "cellular process TM_BIN" BIN
- **isDiagnosisFor**: for concepts assigned to "diagnosis TM_BIN"
- **isClassificationFor:** for concepts assigned to the "epilepsy classification TM_BIN" BIN
- **isImitatorFor:** for concepts assigned to the "epilepsy imitator TM_BIN" BIN
- **isSyndromeFor:** for concepts assigned to the "epilepsy syndrome TM_BIN" BIN
- **isEtiologyFor:** for concepts assigned to the "etiology TM_BIN" BIN**.**
- **isRiksFactorFor**: for concepts assigned to the "risk factor TM_BIN" BIN
- **isseizureClassificationFor**: for concepts assigned to the "seizure classification TM_BIN" BIN
- **isSignandSymptomFor**: for concepts assigned to the "sign and symptom TM_BIN" BIN
- **isTreatmentFor**: for concepts assigned to the "treatment TM_BIN" BIN.

Supplementary text 4: Hierarchical structure of the Epilepsy Ontology (EPIO)

EPIO is a hybrid of multiple OBO Foundry ontology terms and new Epilepsy specific terms. In general, Basic Formal Ontology (BFO) (http://basic-formal-ontology.org/)[1] acts as the top level ontology. Terms from existing Open Biological and Biomedical Ontology (OBO) ontologies such as OBI, OGMS, HP, and GO were integrated by the Ontofox tool[2] in accordance to the OBO Foundry[3] principles and the minimum information to reference an external ontology term (MIREOT) principles[4], whenever possible (Supplementary Figure S1).

EPIO specific terms are often the terminal entries within an ontology hierarchy, comprising the leaves or parents with a short distance to the leaves. Meanwhile, OBO ontologies terms are slightly more central or internal, bridging BFO and EPIO, but they could also be terminal entries (e.g., GO related concepts).

Text Mining related BINS are located in a sidechain outside of the BFO hierarchy. Those concepts enable a collection of search terms for Text Mining applications from different branches. The concepts are related to the Text Mining BINs via specific axioms (relations).

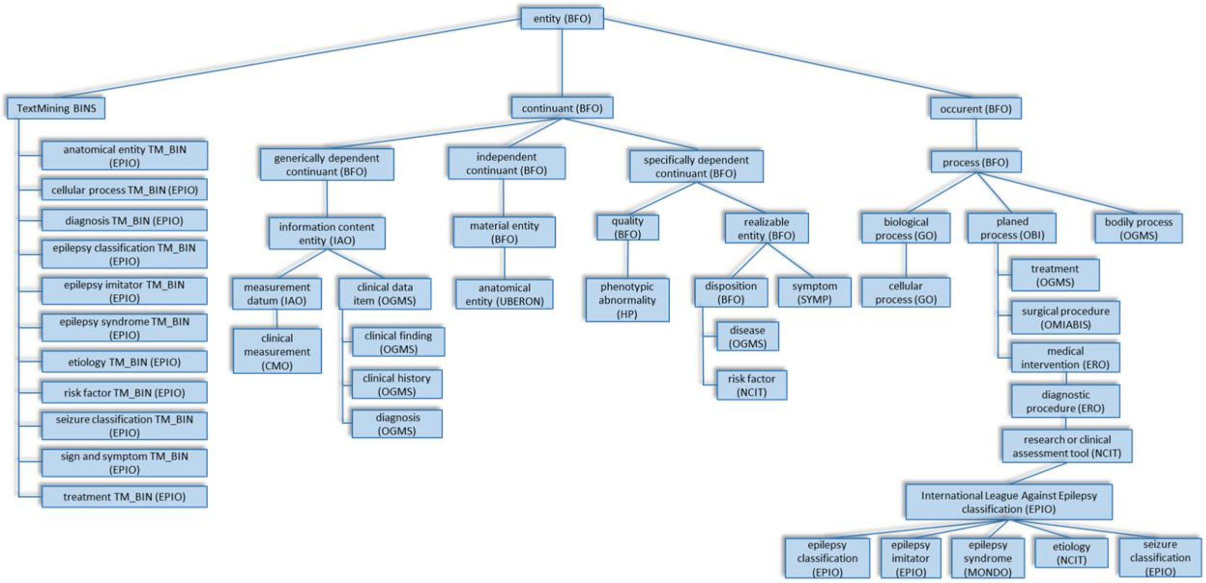

Supplementary Figure 1: Top hierarchical structure of EPIO.

Supplementary text 5: Resources Used to Build The Ontology

- ***International League Against Epilepsy (ILAE)***

In the first stage, all the concepts and definitions provided by International League Against Epilepsy (ILAE) (https://www.epilepsydiagnosis.org/) were used to build the initial version of the EPIO Ontology. ILAE provides a wide knowledge of epilepsy diagnoses and clinical aspects such as epilepsy classification, seizure classification, epilepsy syndromes, epilepsy etiologies and epilepsy imitators, as well as definitions for most of the entities listed on their website. The complete ILAE resource on epilepsy was included in the epilepsy ontology, including its maintained hierarchical structure. In total, 285 entities were imported from ILAE resources. However, there are certain clinical aspects that are not covered in ILAE resources such as screening tests, anatomy, treatment, risk factors, and symptoms. These aspects were collected from other sources.

- ***Epilepsy related ontologies***

Next, concepts from available epilepsy-related ontologies like Epilepsy and Seizure Ontology (https://bioportal.bioontology.org/ontologies/EPSO), ESSO (https://bioportal.bioontology.org/ontologies/ESSO), EPILONT (https://bioportal.bioontology.org/ontologies/EPILONT) were collected. Overall 350 entities from ESSO, 248 from EPSO, 33 from EPILONT were used to extend the initial ILAE-based version described above.

Epilepsy ontology (EPIO) emphasizes the reuse of terms from pre-existing OBO ontologies. Since none of these above mentioned ontologies are in the OBO foundry library (http://obofoundry.org/), priority was given to mapping the concepts to the ones present in any OBO ontologies. Altogether, 1432 entities were imported from existing OBO ontologies and 447 newly defined entities were added to Epilepsy ontology which are not present in any OBO ontology. For each entry, appropriate Epilepsy specific reference links were added.

- ***Publications and web-resources***

Additionally, epilepsy-related knowledge was manually searched in PubMed and specific concepts were added from relevant publications. A list of PMIDs from which concepts were extracted can be found in the table above (520 PubMed articles listed). The number of unique entities imported from different PubMed research articles was 459. 217 concepts were curated from research articles that are not listed in PubMed.

Supplementary text 6: Annotation properties of concepts

A unique primary label (rdfs:label) was added to each concept as a basic annotation, in at least one definition being added to all entities in the Epilepsy ontology. Definitions were always added using the annotation property - oboInOwl:hasDefinition. In accordance with OBO principles, entities and definitions were reused wherever possible. Definitions from ILAE were added to entities when applicable. In case a definition was present in existing ontology and also defined by ILAE, both definitions from both sources were imported to the ontology. Sources of definitions imported from sources other than an existing ontology were sub-annotated with rdfs:isDefinedBy. Database references were added with the annotation oboInOwl:hasDbXRef. Each entity was enhanced with synonyms if those were available.

References

Arp, R., Smith, B., & Spear, A., Building Ontologies with Basic Formal Ontology. (MIT Press, 2015).

Xiang Z, Courtot M, Brinkman RR, Ruttenberg A, He Y. OntoFox: web-based support for ontology reuse. *BMC Research Notes*. 2010, 3:175.

Smith B, Ashburner M, Rosse C, Bard J, Bug W, Ceusters W, et al. The OBO Foundry: coordinated evolution of ontologies to support biomedical data integration. Nat Biotechnol. 2007;25(11):1251–5.

Courtot M, Gibson F, Lister AL, Malone J, Schober D, Brinkman RR, et al. MIREOT: The minimum information to reference an external ontology term. *Applied Ontology*. 2011;6(1):23–33.
