# Supplementary material for: The Epilepsy Ontology: a community-based ontology tailored for semantic interoperability and text mining
Source: Bioinform Adv. 2023 Mar 23;3(1):vbad033. doi: 10.1093/bioadv/vbad033 (PMC10067149; doi:10.1093/bioadv/vbad033)
Supplement: vbad033_Supplementary_Data [file vbad033_supplementary_data.zip › EpilepsyOnt- Supplementary file_FINAL.docx]

**Supplementary Document**

# **Building of the Epilepsy ontology**

## **Supplementary text/table 1: Collection of Concepts**

In order to build the content of the EPIO Ontology, we collected concepts from different sources, including already existing Epilepsy related ontologies (ESSO, Epilepsy and Seizure Ontology, EPILONT), the International League Against Epilepsy (ILAE) website, as well as other domain specific resources and publications. Detailed resource information is described below.

- Initially, 285 concepts and definitions provided by the International League Against Epilepsy (ILAE) website (<https://www.epilepsydiagnosis.org/>) were collected and integrated in the EPIO Ontology.
- Epilepsy related ontologies have been selected and checked. Afterwards, the included concepts have been inspected and new terms were added.
  - Additionally, initial comparison between ontologies was performed before the integration of terms. The results are summarised in the table below:

| **Name** | **Scope** | **Total no. of Concepts** | **No. of Common Concepts** | **No. of Uncommon Concepts** | **Reference** |
| --- | --- | --- | --- | --- | --- |
| EPIO | The Epilepsy Ontology (EPIO) is an assembly of structured knowledge on various aspects of epilepsy, developed according to basic formal ontology (BFO) and Open Biological and Biomedical Ontology (OBO) Foundry principles. Entities and definitions are collected from the latest International League against Epilepsy (ILAE) classification, as well as from domain-specific ontologies such as Epilepsy Ontology (EPILONT), Epilepsy Syndrome Seizure Ontology (ESSO), Epilepsy Semiology(EPISEM) and Epilepsy and Seizure Ontology (EPSO) and scientific literature. | 1916 | - | - | - [http://purl.obolibrary.org/obo/epio.owl](http://purl.obolibrary.org/obo/epso.owl) - [ttps://bioportal.bioontology.org/ontologies/EPIO](https://bioportal.bioontology.org/ontologies/EPIO) |
| ESSO | This ontology contains epilepsy syndromes, seizure types, and data elements associated with them. | 2705 | 356 | 2349 | <https://bioportal.bioontology.org/ontologies/ESSO> |
| Epilepsy and Seizure Ontology | The Epilepsy and Seizure Ontology (EpSO) is an application ontology developed to support epilepsy focused informatics tools for patient care and clinical research. | 1357 | 171 | 1186 | <https://bioportal.bioontology.org/ontologies/EPSO> |
| EPiLONT | Ontology about the epilepsy domain and epileptic seizures. Based on the diagnosis proposed by the International League Against Epilepsy (ILAE). | 138 | 36 | 102 | <https://bioportal.bioontology.org/ontologies/EPILONT> |

**Supplementary Table 3**: Comparison to other related ontologies (as of November 13, 2020)

**Note:** The comparison on concepts was done automatically. This is not very accurate as many of the above mentioned ontologies have not imported concepts from previously existing ontologies or have not re-used the concepts. Therefore, an exact comparison of concept to concept was not feasible.

- Relevant entities and concepts were also collected from 459 research articles. The list of PMIDs of the used articles are mentioned below.

| **Number** | **PMID/DOI** | **Title** |
| --- | --- | --- |
| 1 | 30233290 | In vitro Models for Seizure-Liability Testing Using Induced Pluripotent Stem Cells |
| 2 | 12181003 | Heart rate changes and ECG abnormalities during epileptic seizures: prevalence and definition of an objective clinical sign |
| 3 | 29110774 | Epilepsy |
| 4 | 2384077 | Head circumference in children of epileptic mothers: contributions of drug exposure and genetic background |
| 5 | 25179745 | Obesity and its association with generalised epilepsy, idiopathic syndrome, and family history of epilepsy |
| 6 | 26575850 | Cerebrospinal fluid findings after epileptic seizures |
| 7 | 28288363 | The role of postictal laboratory blood analyses in the diagnosis and prognosis of seizures |
| 8 | 23538271 | Coexistence of epilepsy and Brugada syndrome in a family with SCN5A mutation |
| 9 | 14594442 | Seizures in alcohol-dependent patients: epidemiology, pathophysiology and management |
| 10 | 24441294 | Tobacco smoking, epilepsy, and seizures |
| 11 | 27025991 | [Episodic ataxia type 2 manifests as epileptiform electroencephalographic activity with no epileptic attacks in two family members] |
| 12 | 31327513 | Frequency of takotsubo cardiomyopathy in epilepsy-related hospitalizations among adults and its impact on in-hospital outcomes: A national standpoint |
| 13 | 30691036 | Epilepsy in Children with Autistic Spectrum Disorder |
| 14 | 28775613 | SEIZURE RELATED SEXUAL DISORDER |
| 15 | 29237192 | Pediatric Klüver-Bucy Syndrome: Report of Two Cases and Review of the Literature |
| 16 | 12503648 | Benign familial infantile seizures: further delineation of the syndrome |
| 17 | 15797356 | Educational problems with underlying neuropsychological impairment are common in children with Benign Epilepsy of Childhood with Centrotemporal Spikes (BECTS) |
| 18 | 22916156 | Epilepsy is a risk factor for sudden cardiac arrest in the general population |
| 19 | 20430655 | A patient with DiGeorge syndrome with spina bifida and sacral myelomeningocele, who developed both hypocalcemia-induced seizure and epilepsy |
| 20 | 1831121 | Allergic rash due to antiepileptic drugs: clinical features and management |
| 21 | 26198661 | Two Patients With Visual Aura - Migraine, Epilepsy, or Migralepsy? |
| 22 | 28671982 | Clinical services for adults with an intellectual disability and epilepsy: A comparison of management alternatives |
| 23 | 30562654 | Seizures as a clinical manifestation in somatic autoimmune disorders |
| 24 | 29213881 | Epilepsy in patients with Alzheimer's disease: A systematic review |
| 25 | 11864518 | [Renal failure as a factor leading to epileptic seizures] |
| 26 | 31055731 | Hypertension, seizures, and epilepsy: a review on pathophysiology and management |
| 27 | 11104349 | Association of epilepsy with different groups of microcephaly |
| 28 | 2912820 | Differentiating multiple personality disorder and complex partial seizures |
| 29 | 28749241 | Considerations for ADHD in the child with epilepsy and the child with migraine |
| 30 | 28043687 | Hemiconvulsion-Hemiplegia-Epilepsy Syndrome |
| 31 | 31437864 | Depersonalization- and derealization-like phenomena of epileptic origin |
| 32 | 28360564 | Self-Esteem, Social Phobia and Depression Status in Patients with Epilepsy |
| 33 | 3579680 | Transient global amnesia and epilepsy. Electroencephalographic distinction |
| 34 | 20161538 | Association of epilepsy and comorbid conditions |
| 35 | 30279715 | The Relation between Urinary Tract Infection and Febrile Seizure |
| 36 | 32034533 | Epilepsy in brain metastasis: an emerging entity |
| 37 | 27531853 | [Repeated non-epileptic seizures in a previously healthy young woman - a case report] |
| 38 | 25387857 | Standardized assessment of seizures in patients with juvenile neuronal ceroid lipofuscinosis |
| 39 | 27629553 | Neuronal ceroid lipofuscinoses |
| 40 | 26423537 | Infections, inflammation and epilepsy |
| 41 | 32149989 | Treatment Outcomes of Pediatric Status Epilepticus in a Tertiary Pediatric Intensive Care Unit |
| 42 | 18777476 | Recognition of nonepileptic events |
| 43 | 9532709 | [Epilepsy in patients with ischemic brain disease] |
| 44 | 21741275 | Epilepsy surgery in children with developmental tumours |
| 45 | 15347872 | Spectrum of congenital CNS malformations in pediatric epilepsy |
| 46 | 26478444 | Seizures in oligodendroglial tumors |
| 47 | 31552392 | Zolpidem dependence in an adult with bipolar affective disorder and epilepsy: A case report |
| 48 | 18472483 | Mood disorder and epilepsy: a neurobiologic perspective of their relationship |
| 49 | 32014726 | Long-term follow-up of post-stroke epilepsy after ischemic stroke: Room for improved epilepsy treatment |
| 50 | 30022372 | Serum Uric Acid Is Highly Associated with Epilepsy Secondary to Cerebral Infarction |
| 51 | 31604333 | Preoperative tumor-associated epilepsy in patients with supratentorial meningioma: factors influencing seizure outcome after meningioma surgery |
| 52 | 21851492 | A rare cause of cardiac syncope mimicking epilepsy: left main coronary artery stenosis |
| 53 | 26132164 | Dysgraphia as a Mild Expression of Dystonia in Children with Absence Epilepsy |
| 54 | 6527775 | The role of brain edema in epileptic brain damage induced by systemic kainic acid injection |
| 55 | 31361236 | Pigmented variant of pleomorphic xanthoastrocytoma - A rare long-term epilepsy associated neoplasm |
| 56 | 26362394 | Thyroid hormones: Possible roles in epilepsy pathology |
| 57 | 30924504 | Predictors of Epileptic Seizures and Ability to Work in Supratentorial Cavernous Angioma Located Within Eloquent Brain Areas |
| 58 | 8252282 | Epilepsy and schizophrenia |
| 59 | 19655091 | Myocardial infarction due to late stent thrombosis following epileptic convulsive seizures |
| 60 | 30865078 | Depressive Symptom Severity in Individuals With Epilepsy and Recent Health Complications |
| 61 | 10474720 | HIV infection and seizures |
| 62 | 27856781 | Parental rheumatoid arthritis and childhood epilepsy: A nationwide cohort study |
| 63 | 20301555 | Aicardi Syndrome |
| 64 | 26549780 | Comorbidities of epilepsy: current concepts and future perspectives |
| 65 | 29379967 | Development of Neuromyelitis Optica Spectrum Disorder and Spinal Arachnoid Cysts in a Patient With Intractable Epilepsy |
| 66 | 24687183 | Population-level evidence for an autoimmune etiology of epilepsy |
| 67 | 24073547 | Epilepsy in children with subacute sclerosing panencephalitis |
| 68 | 25214788 | Risk of epilepsy in patients with systemic lupus erythematosus - a retrospective cohort study |
| 69 | 29791879 | Seizure imitators monitored using video-EEG in children with intellectual disabilities |
| 70 | 29241678 | Language Dysfunction in Pediatric Epilepsy |
| 71 | 29720810 | Recovery of Visual Scotomas by Vortioxetine in a Patient with Symptomatic Occipital Lobe Epilepsy |
| 72 | 30600130 | Epilepsy in children with type 1 diabetes mellitus: Pathophysiological basis and clinical hallmarks |
| 73 | 26468872 | Delayed Intraparenchymal Hematoma Following Diagnostic Lumbar Puncture |
| 74 | 30661434 | From next-generation sequencing to targeted treatment of non-acquired epilepsies |
| 75 | 27430454 | Neuroimaging of epilepsy |
| 76 | 27789166 | How neuropsychology can improve the care of individual patients with epilepsy. Looking back and into the future |
| 77 | 21490734 | Role of single photon emission computed tomography in epilepsy |
| 78 | 29722352 | Epilepsy |
| 79 | 14537108 | Positron emission tomography and epilepsy |
| 80 | 22554135 | The effect of seizure focus on regional language processing areas |
| 81 | 17855377 | Stereoelectroencephalography in presurgical assessment of MRI-negative epilepsy |
| 82 | 26721354 | Epileptogenic effects of G protein-coupled estrogen receptor 1 in the rat pentylenetetrazole kindling model of epilepsy |
| 83 | 28089585 | The impact of intelligence on memory and executive functions of children with temporal lobe epilepsy: Methodological concerns with clinical relevance |
| 84 | 18976727 | A homozygous mutation in human PRICKLE1 causes an autosomal-recessive progressive myoclonus epilepsy-ataxia syndrome |
| 85 | 2782045 | Eating epilepsy |
| 86 | 17371290 | Apoptosis signalling pathways in seizure-induced neuronal death and epilepsy |
| 87 | 29961525 | Focal Epilepsy in a Teenager With Facial Atrophy and Hair Loss |
| 88 | 12365699 | Unusual eye movements in a patient with complex partial seizure disorder |
| 89 | 8441366 | Behavioral changes associated with epilepsy |
| 90 | 28139515 | Clinical Analysis of Partial Epilepsy with Auras |
| 91 | 20301709 | Autosomal Dominant Epilepsy with Auditory Features |
| 92 | 17260039 | Depression and epilepsy: a new perspective on two closely related disorders |
| 93 | 13365719 | A case of precocious puberty with epileptic seizure |
| 94 | 30838190 | Precocious and Early Central Puberty in Children With Pre-existing Medical Conditions: A Single Center Study |
| 95 | 29172092 | Visual field defects after temporal lobe resection for epilepsy |
| 96 | 31603835 | [Epilepsy in children with congenital hemiparesis secondary to perinatal ictus] |
| 97 | 27188686 | Cerebral palsy |
| 98 | 2761703 | Seizures after primary intracerebral hemorrhage |
| 99 | 26864574 | When the face says it all: dysmorphology in identifying syndromic causes of epilepsy |
| 100 | 30118929 | Ictal nausea and vomiting - Is it left or right? |
| 101 | 30125861 | The role of magnetoencephalography in the presurgical evaluation of patients with MRI-negative operculo-insular epilepsy |
| 102 | 12199726 | Interictal EEG abnormalities in patients with psychogenic nonepileptic seizures |
| 103 | 29656099 | Hypsarrhythmia in epileptic spasms: Synchrony in chaos |
| 104 | 30378543 | Sleep-related hypermotor epilepsy and peri-ictal hypotension in a patient with syntaxin-1B mutation |
| 105 | 30666028 | Valproic acid-induced nocturnal enuresis in pediatric patients |
| 106 | 15642493 | Symptoms in focal sensory seizures. Clinical and electroencephalographic features |
| 107 | 27857611 | Epilepsy and the Sensory Systems |
| 108 | 10919145 | [Anatomic substrate of epigastric aura: case report] |
| 109 | 15911361 | Somatosensory auras in focal epilepsy: a clinical, video EEG and MRI study |
| 110 | 26249726 | The relevance of somatosensory auras in refractory temporal lobe epilepsies |
| 111 | 12134331 | [Epileptic auras: classification, pathophysiology, practical usefulness, differential diagnosis and controversials] |
| 112 | 12609279 | Integration of Perceptual and Mnemonic Dysfunction: Sensory Auras Are Associated with Left Hemispheric Memory Impairment |
| 113 | 30068811 | [A case of painful seizure accompanying ictal paresis and homonymous hemianopia due to post-stroke epilepsy] |
| 114 | 22791548 | Provider practices impact adequate diagnosis of sleep disorders in children with epilepsy |
| 115 | 30661063 | Brain Morphology in Patients with Genetic Generalized Epilepsy: Its Heterogeneity among Subsyndromes |
| 116 | 28488762 | Visual Auras in Epilepsy and Migraine - An Analysis of Clinical Characteristics |
| 117 | 23365482 | Vasovagal syncope treated as epilepsy for 16 years |
| 118 | 20627778 | Partial epilepsy presenting as focal atonic seizure: a case report |
| 119 | 6820630 | [Tonic epileptic crisis and complex and mixed atypical absences in Lennox-Gastaut syndrome in patients over 6 years of age] |
| 120 | 22580903 | Obstructive sleep apnea and primary snoring in children with epilepsy |
| 121 | 22776676 | Complex epileptic palilalia: a case report |
| 122 | 26088882 | Effect of epileptic seizures on the cerebrospinal fluid--A systematic retrospective analysis |
| 123 | 22844307 | Focal cortical dysplasia - review |
| 124 | 31085954 | Pathological Classification of Focal Cortical Dysplasia (FCD) : Personal Comments for Well Understanding FCD Classification |
| 125 | 30711777 | Parasites and epilepsy: Understanding the determinants of epileptogenesis |
| 126 | 19744116 | Nonconvulsive status epilepticus and coma |
| 127 | 26336950 | A definition and classification of status epilepticus--Report of the ILAE Task Force on Classification of Status Epilepticus |
| 128 | 26920416 | Treatment of Convulsive Status Epilepticus |
| 129 | 24977129 | Recurrent seizures following focal motor status epilepticus in a patient with non-ketotic hyperglycemia and acute cerebral infarction |
| 130 | 7469854 | Acute epidural hematoma following epileptic seizures |
| 131 | 25905906 | Cognitive impairment in epilepsy: the role of network abnormalities |
| 132 | 25856437 | Revealing Medicinal Plants That Are Useful for the Comprehensive Management of Epilepsy and Associated Comorbidities through In Silico Mining of Their Phytochemical Diversity |
| 133 | 30473971 | Postictal Mania Versus Postictal Psychosis |
| 134 | 9579937 | Dissociation in epilepsy and conversion nonepileptic seizures |
| 135 | 16650147 | Epileptic seizures superimposed on catatonic stupor |
| 136 | 9291731 | Formal thought disorder and psychopathology in pediatric primary generalized and complex partial epilepsy |
| 137 | 26167207 | Assessing the prevalence distribution of abnormal laboratory tests in patients with simple febrile seizure |
| 138 | 31911412 | Transient seizure-induced sodium increase camouflaging a symptomatic hyponatremia |
| 139 | 23534590 | [Cerebral creatine deficiency syndromes] |
| 140 | 25777785 | Major congenital malformations in children of women with epilepsy |
| 141 | 11076003 | Venous angiomas and epilepsy |
| 142 | 24134485 | Cavernoma-related epilepsy: review and recommendations for management--report of the Surgical Task Force of the ILAE Commission on Therapeutic Strategies |
| 143 | 31578307 | Activity of hippocampal adult-born neurons regulates alcohol withdrawal seizures |
| 144 | 30782577 | A novel mutation in KCNQ3-related benign familial neonatal epilepsy: electroclinical features and neurodevelopmental outcome |
| 145 | 2512371 | Epileptic seizures in intracerebral haemorrhage |
| 146 | 15146004 | Clinical features and long term outcome of epilepsy in periventricular nodular heterotopia. Simple compared with plus forms |
| 147 | 28658095 | A clinical review on megalencephaly: A large brain as a possible sign of cerebral impairment |
| 148 | 31830676 | New-onset refractory status epilepticus: A retrospective cohort study |
| 149 | 32062735 | Novel mutation of SCN9A gene causing generalized epilepsy with febrile seizures plus in a Chinese family |
| 150 | 28671587 | Inborn Errors of Metabolism and Epilepsy: Current Understanding, Diagnosis, and Treatment Approaches |
| 151 | 26592968 | Postencephalitic epilepsy and drug-resistant epilepsy after infectious and antibody-associated encephalitis in childhood: Clinical and etiologic risk factors |
| 152 | 6955943 | Attentional and perceptual disturbances in children with Tourette's syndrome, attention deficit disorder, and epilepsy |
| 153 | 28116304 | Alice in Wonderland Syndrome: A Clinical and Pathophysiological Review |
| 154 | 8500435 | Epilepsy in the setting of neurocutaneous syndromes |
| 155 | 24761136 | Post-traumatic epilepsy: an overview |
| 156 | 23551133 | Complications of epilepsy surgery: a systematic review of focal surgical resections and invasive EEG monitoring |
| 157 | 32038177 | The Epilepsy of Infancy With Migrating Focal Seizures: Identification of de novo Mutations of the KCNT2 Gene That Exert Inhibitory Effects on the Corresponding Heteromeric K Na 1.1/K Na 1.2 Potassium Channel |
| 158 | 25917466 | Febrile seizures and genetic epilepsy with febrile seizures plus (GEFS+) |
| 159 | 21135885 | The role of inflammation in epilepsy |
| 160 | 17984449 | Obstructive sleep apnea is associated with seizure occurrence in older adults with epilepsy |
| 161 | 26441491 | Modulation of autonomic activity in neurological conditions: Epilepsy and Tourette Syndrome |
| 162 | 20696621 | Hormonal alterations following seizures |
| 163 | 25966854 | Asystole in the epilepsy unit |
| 164 | 26394714 | Genotype-phenotype correlation of congenital anomalies in multiple congenital anomalies hypotonia seizures syndrome (MCAHS1)/PIGN-related epilepsy |
| 165 | 27768938 | Hyperammonaemia and associated factors in unprovoked convulsive seizures: A cross-sectional study |
| 166 | 25180909 | Causes and consequences of gray matter heterotopia |
| 167 | 7487261 | Value of tongue biting in the diagnosis of seizures |
| 168 | 29414563 | Genital automatisms: Reappraisal of a remarkable but ignored symptom of focal seizures |
| 169 | 16103019 | The role of serotonin in impulsive and aggressive behaviors associated with epilepsy-like neuronal hyperexcitability in the amygdala |
| 170 | 29287215 | Impact of vagus nerve stimulation on sleep-related breathing disorders in adults with epilepsy |
| 171 | 10996566 | NREM parasomnias: arousal disorders and differentiation from nocturnal frontal lobe epilepsy |
| 172 | 19162231 | Focal epileptic seizures mimicking sleep paralysis |
| 173 | 32086099 | Focal cortical hypermetabolism in atypical benign rolandic epilepsy |
| 174 | 21633606 | Management of provoked seizure |
| 175 | 31617494 | Intermittent photic stimulation-provoked seizure associated with ictal asystole |
| 176 | 29325826 | Myoclonic absence seizures with complex gestural automatisms |
| 177 | 31832260 | High-dose Clozapine Withdrawal: A Case Report and Timeline of a Single Potential Withdrawal Seizure |
| 178 | 15528919 | A note on gelastic epilepsy |
| 179 | 29170920 | Dacrystic Epilepsy |
| 180 | 22938964 | Animal models |
| 181 | 28332054 | A Mesiotemporal Lobe Epilepsy Mouse Model |
| 182 | 29933054 | Imaging correlates of behavioral impairments: An experimental PET study in the rat pilocarpine epilepsy model |
| 183 | 23929939 | Modeling human neurodevelopmental disorders in the Xenopus tadpole: from mechanisms to therapeutic targets |
| 184 | 13268119 | Epilepsy in Macaca mulatta after cortical or intracerebral alumina |
| 185 | 30219655 | Circadian and circaseptan rhythms in human epilepsy: a retrospective cohort study |
| 186 | 23190285 | Common imitators of epilepsy |
| 187 | 24679945 | The utility score of epilepsy with partial seizure measured by TTO, VAS, and EQ-5D in the general Korean population |
| 188 | 22512895 | A comparison of quality of life in adolescents with epilepsy or asthma using the Short-Form Health Survey (SF-36) |
| 189 | 11081816 | Daytime sleepiness in epilepsy patients: evaluation by means of the Epworth sleepiness scale |
| 190 | 28064112 | Ultra-short screening instruments for major depressive episode and generalized anxiety disorder in epilepsy: The NDDIE-2 and the GAD-SI |
| 191 | 30599368 | Hospital Anxiety and Depression Scale-Anxiety subscale (HADS-A) and The State-Trait Anxiety Inventory (STAI) accuracy for anxiety disorders detection in drug-resistant mesial temporal lobe epilepsy patients |
| 192 | 31026785 | Reliability and validity of the Chinese version of the Patient Health Questionnaire 9 (C-PHQ-9) in patients with epilepsy |
| 193 | 26921599 | Associations of impaired sleep quality, insomnia, and sleepiness with epilepsy: A questionnaire-based case-control study |
| 194 | 21576040 | Self-reported medication adherence and treatment satisfaction in patients with epilepsy |
| 195 | 26255305 | Diffusion MRI and its Role in Neuropsychology |
| 196 | 27914224 | A systematic review of epileptic seizures in adults with subdural haematomas |
| 197 | 25204010 | Intraoperative ElectroCorticoGraphy (ECog): indications, techniques, and utility in epilepsy surgery |
| 198 | 31914330 | The adverse-effect profile of lacosamide |
| 199 | 26874864 | Interictal epileptiform discharge effects on neuropsychological assessment and epilepsy surgical planning |
| 200 | 23941480 | Occupational therapy and epilepsy |
| 201 | 31461681 | Usefulness of preschool and school versions of the Behavioral Rating Inventory of Executive Functions in the evaluation of the daily life executive function in myoclonic-atonic epilepsy |
| 202 | 22258041 | Montreal Cognitive Assessment in cryptogenic epilepsy patients with normal Mini-Mental State Examination scores |
| 203 | 29991426 | Positive Airway Pressure Therapy Is Challenging for Patients With Epilepsy |
| 204 | 21849000 | Effect of continuous positive airway pressure treatment on seizure control in patients with obstructive sleep apnea and epilepsy |
| 205 | 27101469 | The role of MEG in pre-surgical evaluation of epilepsy: current use and future directions |
| 206 | 30819542 | Autonomic biofeedback therapy in epilepsy |
| 207 | 20345933 | Alterations of intracerebral γ-aminobutyric acid (GABA) levels by titration with levetiracetam in patients with focal epilepsies |
| 208 | 24861650 | The preoperative evaluation and surgical treatment of epilepsy |
| 209 | 29140112 | A 2017 review of pharmacotherapy for treating focal epilepsy: where are we now and how will treatment develop? |
| 210 | 27091679 | Descriptive study of symptomatic epilepsy by age of onset in patients with a 3-year follow-up at the Neuropaediatric Department of a reference centre |
| 211 | 30909075 | Default mode network deactivation in pediatric temporal lobe epilepsy: Relationship to a working memory task and executive function tests |
| 212 | 28324301 | Neuroimaging in Epilepsy |
| 213 | 15281961 | Magnetoencephalography in epilepsy |
| 214 | 8215194 | Computed tomography in patients with recurrent seizures |
| 215 | 23250841 | Critical review of palliative surgical techniques for intractable epilepsy |
| 216 | 21269290 | Epilepsy and the new cytogenetics |
| 217 | 30086482 | In vitro and in vivo experimental models employed in the discovery and development of antiepileptic drugs for pharmacoresistant epilepsy |
| 218 | 20492865 | [Experimental models in epilepsy] |
| 219 | 30030085 | Deep brain stimulation for epilepsy |
| 220 | 28384785 | Maternal Body Mass Index in Early Pregnancy and Risk of Epilepsy in Offspring |
| 221 | 27210239 | Psychiatric and Behavioural Disorders in Children with Epilepsy: an ILAE Task Force Report |
| 222 | 16380232 | Epilepsy, surgery, and the elderly |
| 223 | 29111504 | Validation of the Polish version of the Beck Depression Inventory in patients with epilepsy |
| 224 | 29624147 | Risk factors for surgical site infection after intracranial electroencephalography monitoring for epilepsy in the pediatric population |
| 225 | 29382476 | [Factors associated with epilepsy in children in Mexico: A case-control study] |
| 226 | 19243383 | Global expression profiling in epileptogenesis: does it add to the confusion? |
| 227 | 28382495 | Cognitive and Behavioral Interventions in Epilepsy |
| 228 | 30591281 | Laser interstitial thermotherapy (LiTT) in pediatric epilepsy surgery |
| 229 | 21909104 | Glutamate release by primary brain tumors induces epileptic activity |
| 230 | 25819950 | Classical neurotransmitters and neuropeptides involved in generalized epilepsy in a multi-neurotransmitter system: How to improve the antiepileptic effect? |
| 231 | 25487080 | Antiepileptic drugs influences on body weight in people with epilepsy |
| 232 | 28858722 | Comprehension and production of nouns and verbs in temporal lobe epilepsy |
| 233 | 26293325 | When patients with epilepsy or "epilepsy" might need a pacemaker |
| 234 | 22693291 | Progressive seizures in a patient with congenital coagulopathies |
| 235 | 25260205 | Idiopathic brain herniation. A report of two paediatric cases |
| 236 | 29888583 | [A Meta-analysis of the Effectiveness of Acupuncture in the Treatment of Epilepsy] |
| 237 | 26033084 | Seizures and epilepsy: an overview for neuroscientists |
| 238 | 28190753 | Evaluation of the first seizure patient: Key points in the history and physical examination |
| 239 | 29107258 | Epilepsy: Clinical Review and Surgical Options |
| 240 | 28951736 | Classification of Hand Grasp Kinetics and Types Using Movement-Related Cortical Potentials and EEG Rhythms |
| 241 | 23515147 | Dietary therapies for epilepsy |
| 242 | 15608956 | Intelligence functions disorders in patients with complex partial epilepsy |
| 243 | 2871721 | Kindling model of epilepsy |
| 244 | 16059497 | Sleep deprivation and epilepsy |
| 245 | 3064627 | Theta rhythms in the EEG: a genetic trait in childhood epilepsy |
| 246 | 10908505 | Yoga for epilepsy |
| 247 | 13789853 | Traumatic epilepsy after closed head injuries |
| 248 | 14630489 | Use of aromatherapy (with or without hypnosis) in the treatment of intractable epilepsy--a two-year follow-up study |
| 249 | 26835338 | Hemispherectomy in the treatment of seizures: a review |
| 250 | 30760973 | Ketogenic Diet and Epilepsy: What We Know So Far |
| 251 | 22826811 | Vagus nerve stimulation for epilepsy: A review of the peripheral mechanisms |
| 252 | 28214547 | Cerebral small vessel disease predisposes to temporal lobe epilepsy in spontaneously hypertensive rats |
| 253 | 24686330 | Slow modulations of high-frequency activity (40-140-Hz) discriminate preictal changes in human focal epilepsy |
| 254 | 28532712 | Photosensitivity and epilepsy: Current concepts and perspectives-A narrative review |
| 255 | 32154929 | Peri-ictal responsiveness to the social environment is greater in psychogenic nonepileptic than epileptic seizures |
| 256 | 26677173 | Inhibition of p38 mitogen-activated protein kinase signaling reduces multidrug transporter activity and anti-epileptic drug resistance in refractory epileptic rats |
| 257 | 27346214 | Immune response in the eye following epileptic seizures |
| 258 | 30912766 | Pro-inflammatory, IL-17 pathways dominate the architecture of the immunome in pediatric refractory epilepsy |
| 259 | 29467619 | Cannabidiol Regulates Long Term Potentiation Following Status Epilepticus: Mediation by Calcium Stores and Serotonin |
| 260 | 30767241 | New insights into human lysine degradation pathways with relevance to pyridoxine-dependent epilepsy due to antiquitin deficiency |
| 261 | 23252947 | Association of carbamazepine major metabolism and transport pathway gene polymorphisms and pharmacokinetics in patients with epilepsy |
| 262 | 24114605 | Atypical vitamin B6 deficiency: a rare cause of unexplained neonatal and infantile epilepsies |
| 263 | 29359340 | Review: Mechanistic target of rapamycin (mTOR) pathway, focal cortical dysplasia and epilepsy |
| 264 | 29737480 | Notch Signaling Regulates Microglial Activation and Inflammatory Reactions in a Rat Model of Temporal Lobe Epilepsy |
| 265 | 26260962 | Epilepsy and innate immune system: A possible immunogenic predisposition and related therapeutic implications |
| 266 | 24914213 | Protein degradation and quality control in cells from laforin and malin knockout mice |
| 267 | 29512697 | Ephrin‑b3 modulates hippocampal neurogenesis and the reelin signaling pathway in a pilocarpine‑induced model of epilepsy |
| 268 | 30262417 | Brain-derived neurotrophic factor and epilepsy: a systematic review |
| 269 | 17910583 | Alterations of phosphatidylinositol 3-kinase pathway components in epilepsy-associated glioneuronal lesions |
| 270 | 25221392 | Pharmacogenetic evaluation of ABCB1, Cyp2C9, Cyp2C19 and methylene tetrahydrofolate reductase polymorphisms in teratogenicity of anti-epileptic drugs in women with epilepsy |
| 271 | 25433904 | Altered glutamate protein co-expression network topology linked to spine loss in the auditory cortex of schizophrenia |
| 272 | 28074534 | Depolarizing γ-aminobutyric acid contributes to glutamatergic network rewiring in epilepsy |
| 273 | 24723228 | Calcium signaling and epilepsy |
| 274 | 26254980 | Genetically epilepsy-prone rats (GEPRs) and DBA/2 mice: Two animal models of audiogenic reflex epilepsy for the evaluation of new generation AEDs |
| 275 | 25312505 | Advances on genetic rat models of epilepsy |
| 276 | 28506440 | The Wistar Audiogenic Rat (WAR) strain and its contributions to epileptology and related comorbidities: History and perspectives |
| 277 | 11219629 | Hydrocephalus and epilepsy |
| 278 | 25535236 | Skin lesions in a patient with epilepsy |
| 279 | 30416962 | Recurrent focal seizures as a feature of status epilepticus presenting as a peri-ictal water drinking |
| 280 | 22698381 | Prolonged atrial fibrillation following generalized tonic-clonic seizures |
| 281 | 22637287 | Atrial fibrillation associated with epileptic seizures |
| 282 | 18539570 | Unilateral mydriasis during temporal lobe seizures |
| 283 | 30698542 | [Anxiety disorders in epilepsy] |
| 284 | 23142708 | The diagnostic value of urinary incontinence in the differential diagnosis of seizures |
| 285 | 27638925 | Mechanisms of memory impairment in epilepsy depend on age at disease onset |
| 286 | 29763181 | Simple Partial Seizure |
| 287 | 18754955 | Bacterial meningitis and epilepsy |
| 288 | 15562299 | Effects of Seizures on Autonomic and Cardiovascular Function |
| 289 | 23613463 | Low-frequency electrical stimulation of a fiber tract in temporal lobe epilepsy |
| 290 | 23671345 | Subcortical epilepsy? |
| 291 | 23622192 | Brain maturation and epilepsy |
| 292 | 5096551 | Focal dysplasia of the cerebral cortex in epilepsy |
| 293 | 3987648 | Effect of midbrain and pontine tegmental lesions on audiogenic seizures in genetically epilepsy-prone rats |
| 294 | 23810707 | Midbrain-hindbrain malformations in patients with malformations of cortical development and epilepsy: a series of 220 patients |
| 295 | 26368332 | Olfactory Bulbectomy Leads to the Development of Epilepsy in Mice |
| 296 | 22091816 | Ventricular enlargement in new-onset pediatric epilepsies |
| 297 | 28912749 | Functional Connectivity of the Corpus Callosum in Epilepsy Patients with Secondarily Generalized Seizures |
| 298 | 31920906 | Superior Frontal Sulcus Focal Cortical Dysplasia Type II: An MRI, PET, and Quantified SEEG Study |
| 299 | 26063964 | The Classical Pathways of Occipital Lobe Epileptic Propagation Revised in the Light of White Matter Dissection |
| 300 | 15857433 | Entorhinal cortex involvement in human mesial temporal lobe epilepsy: an electrophysiologic and volumetric study |
| 301 | 30269938 | Distinctive epileptogenic networks for parietal operculum seizures |
| 302 | 21403025 | Cingulate gyrus epilepsy: clinical and behavioral aspects, with surgical outcomes |
| 303 | 30100562 | Cingulate gyrus epilepsy |
| 304 | 28928072 | Orbitofrontal epilepsy: Case series and review of literature |
| 305 | 21925841 | EEG background activity is abnormal in the temporal and inferior parietal cortex in benign rolandic epilepsy of childhood: a LORETA study |
| 306 | 26295917 | A case of secondary somatosensory epilepsy with a left deep parietal opercular lesion: successful tumor resection using a transsubcentral gyral approach during awake surgery |
| 307 | 10775518 | Quantitative MRI volumetry of the entorhinal cortex in temporal lobe epilepsy |
| 308 | 29519472 | Parietal lobe epilepsy |
| 309 | 11377839 | Evidence of peripheral auditory activity modulation by the auditory cortex in humans |
| 310 | 18209276 | Depression, hopelessness and suicide risk among patients suffering from epilepsy |
| 311 | 29960852 | Improving compliance in adults with epilepsy on a modified Atkins diet: A randomized trial |
| 312 | 11684349 | Expression of vesl-1S/homer-1a, a gene associated with long-term potentiation, in the brain of the epileptic EI mouse |
| 313 | 1834893 | A slow intravenous infusion of N-methyl-DL-aspartate as a seizure model in the mouse |
| 314 | 25568300 | The phenotypic spectrum of SCN8A encephalopathy |
| 315 | 31044310 | Comparing the Wada Test and Functional MRI for the Presurgical Evaluation of Memory in Temporal Lobe Epilepsy |
| 316 | 25228809 | Animal models of epilepsy: use and limitations |
| 317 | 25845493 | Cross-species pharmacological characterization of the allylglycine seizure model in mice and larval zebrafish |
| 318 | 21532379 | Frontal lobe seizures |
| 319 | 9932952 | Anterior temporal abnormality in temporal lobe epilepsy: a quantitative MRI and histopathologic study |
| 320 | 30714986 | Autoimmune seizures and epilepsy |
| 321 | 30139784 | Ictal cardiorespiratory depression: a real risk for sudden unexpected death in epilepsy (SUDEP)? |
| 322 | 28324947 | A sparse Laguerre-Volterra autoregressive model for seizure prediction in temporal lobe epilepsy |
| 323 | 2345619 | Characterization of the basal temporal language area in patients with left temporal lobe epilepsy |
| 324 | 20231918 | Benign EEG patterns: is there more to learn? |
| 325 | 11292215 | Benign myoclonus of early infancy: an imitator of West's syndrome |
| 326 | 15032388 | Benign neonatal sleep myoclonus mimicking status epilepticus |
| 327 | 16302879 | Nonepileptic disorders imitating generalized idiopathic epilepsies |
| 328 | 1396544 | Chemical models of epilepsy with some reference to their applicability in the development of anticonvulsants |
| 329 | 29623857 | The Large Conductance Calcium- and Voltage-activated Potassium Channel (BK) and Epilepsy |
| 330 | 1283140 | Effects of antiepileptic drugs, calcium channel blockers and other compounds on seizures induced by activation of voltage-dependent L calcium channel in DBA/2 mice |
| 331 | 15941650 | Benign childhood epilepsy with occipital paroxysms: neuropsychological findings |
| 332 | 9024190 | Functional anatomy of spontaneous seizures in a rat model of limbic epilepsy |
| 333 | 10802766 | Anterior cingulate gyrus epilepsy: the role of ictal rCBF SPECT in seizure localization |
| 334 | 30528098 | Conventional and quantitative EEG in status epilepticus |
| 335 | 25769270 | Animal models of temporal lobe epilepsy following systemic chemoconvulsant administration |
| 336 | 10510977 | Corneal kindling in mice: behavioral and pharmacological differences to conventional kindling |
| 337 | 22995680 | Computational models of epilepsy |
| 338 | 23027094 | Dorsolateral frontal lobe epilepsy |
| 339 | 25687591 | EEG guidelines in the diagnosis of brain death |
| 340 | 24405074 | Stereotactic placement of depth electrodes in medically intractable epilepsy |
| 341 | 27093945 | Extrastriate visual cortex in idiopathic occipital epilepsies: The contribution of retinotopic areas to spike generation |
| 342 | 7925156 | Extratemporal cortical resections and lesionectomies for partial epilepsy: complications of surgical treatment |
| 343 | 17162190 | Ictal EEG in benign partial epilepsy in infancy |
| 344 | 30057567 | Clinical Management of Epilepsy With Glutamic Acid Decarboxylase Antibody Positivity: The Interplay Between Immunotherapy and Anti-epileptic Drugs |
| 345 | 20868357 | In vivo experimental models of epilepsy |
| 346 | 17525024 | Focal motor seizure with automatisms in a newborn |
| 347 | 16563807 | Cognitive problems related to epilepsy syndromes, especially malignant epilepsies |
| 348 | 23667393 | Dissociative seizures: a challenge for neurologists and psychotherapists |
| 349 | 24649451 | Simple Partial Status of Forced Thinking Originated in the Mesial Temporal Region: Intracranial Foramen Ovale Electrode Recording and Ictal PET |
| 350 | 22832396 | Cognitive dysfunction with complex visual hallucinations due to focal nonconvulsive status epilepticus: a neuropsychological study and SISCOM |
| 351 | 29376090 | Cognitive impairment in epilepsy: the role of reduced network flexibility |
| 352 | 19846832 | Directed aggressive behavior in frontal lobe epilepsy: a video-EEG and ictal spect case study |
| 353 | 26924970 | Ecstatic Epileptic Seizures: A Glimpse into the Multiple Roles of the Insula |
| 354 | 30361137 | Movement disorders phenomenology in focal motor seizures |
| 355 | 8848969 | Epileptic negative myoclonus |
| 356 | 19679190 | Evidence that juvenile myoclonic epilepsy is a disorder of frontotemporal corticothalamic networks |
| 357 | 11554899 | Gait epilepsy. A case report of gait-induced seizures |
| 358 | 29327337 | Generalized nonmotor (absence) seizures-What do absence, generalized, and nonmotor mean? |
| 359 | 19423297 | Delusions, illusions and hallucinations in epilepsy: 1. Elementary phenomena |
| 360 | 8021672 | Heautoscopy, epilepsy, and suicide |
| 361 | 24777136 | Epilepsy and the hippocampus |
| 362 | 27070861 | Effects of hippocampal partial kindling on sensory and sensorimotor gating and methamphetamine-induced locomotion in kindling-prone and kindling-resistant rats |
| 363 | 21030341 | Hypnopompic seizures |
| 364 | 26038597 | Cardiac arrhythmias during or after epileptic seizures |
| 365 | 26304794 | Ictal pattern on scalp EEG at onset of seizure in temporal lobe epilepsy: Old and new problems for epileptologists |
| 366 | 15329073 | Clinical manifestations of insular lobe seizures: a stereo-electroencephalographic study |
| 367 | 28637636 | Epilepsy after cerebral infection: review of the literature and the potential for surgery |
| 368 | 12744361 | Complications of invasive subdural grid monitoring in children with epilepsy |
| 369 | 6149233 | The jittery newborn and infant: a review |
| 370 | 19286474 | Gelastic seizures: A case of lateral frontal lobe epilepsy and review of the literature |
| 371 | 15788545 | Occipital epilepsy: lateral versus mesial |
| 372 | 23872083 | Hypermotor seizures in lateral and mesial parietal epilepsy |
| 373 | 26317672 | Neurocognitive Changes Associated With Surgical Resection of Left and Right Temporal Lobe Glioma |
| 374 | 21896690 | Mesial temporal sclerosis in epilepsy |
| 375 | 31846897 | Low CSF CD4/CD8+ T-cell proportions are associated with blood-CSF barrier dysfunction in limbic encephalitis |
| 376 | 25667835 | Ictal kissing with subdural EEG recording |
| 377 | 1324090 | Ontogenic study of lithium-pilocarpine-induced status epilepticus in rats |
| 378 | 23027093 | Mesial frontal lobe epilepsy |
| 379 | 23027091 | Mesial temporal lobe epilepsy |
| 380 | 6133913 | Alterations in the content of amino acid neurotransmitters before the onset and during the course of methoxypyridoxine-induced seizures in individual rabbit brain regions |
| 381 | 24632481 | Study of psychiatric comorbidities in epilepsy by using the Mini International Neuropsychiatric Interview |
| 382 | 20627816 | The diagnostic utility of the ictal cry |
| 383 | 1915173 | Benign partial epilepsy of childhood with monomorphic sharp waves in centrotemporal and other locations |
| 384 | 9757434 | Multilobar resections in surgical treatment of medically intractable epilepsy |
| 385 | 8194189 | Multiple sleep latency tests in epilepsy |
| 386 | 12908748 | Negative and positive visual hypnotic hallucinations: attending inside and out |
| 387 | 23531441 | Head atonic attacks: a new type of benign non-epileptic attack in infancy strongly mimicking epilepsy |
| 388 | 2369876 | Differentiation of epileptic from nonepileptic head drops in children |
| 389 | 9109891 | Noninvasive assessment of language dominance in children and adolescents with functional MRI: a preliminary study |
| 390 | 9832214 | Non-invasive assessment of language dominance with near-infrared spectroscopic mapping |
| 391 | 7631075 | Role of multichannel magnetoencephalography in the evaluation of ablative seizure surgery candidates |
| 392 | 27627857 | Non-invasive Evaluation for Epilepsy Surgery |
| 393 | 24163755 | NON-INVASIVE BRAIN STIMULATION IN CHILDREN: APPLICATIONS AND FUTURE DIRECTIONS |
| 394 | 12684556 | Clinical correlation of occipital intermittent rhythmic delta activity |
| 395 | 15489401 | Visual disturbances representing occipital lobe epilepsy in patients with cerebral calcifications and coeliac disease: a case series |
| 396 | 23027097 | Occipital lobe seizures and epilepsies |
| 397 | 9128446 | Occipital pole area and language dominance |
| 398 | 835966 | Ocular oscillations |
| 399 | 2909707 | Oligoantigenic diet treatment of children with epilepsy and migraine |
| 400 | 24881594 | Prevention of organophosphate-induced chronic epilepsy by early benzodiazepine treatment |
| 401 | 23384238 | The use of organotypic slice cultures for the study of epileptogenesis |
| 402 | 24592228 | Out-of-body experiences associated with seizures |
| 403 | 19453720 | Parahippocampal epilepsy with subtle dysplasia: A cause of "imaging negative" partial epilepsy |
| 404 | 30909077 | The neuropsychological profile of parietal and occipital lobe epilepsy |
| 405 | 2779593 | The sz mutant hamster: a genetic model of epilepsy or of paroxysmal dystonia? |
| 406 | 29151098 | The Alice-in-Wonderland Syndrome |
| 407 | 18762233 | Penicillin-induced epilepsy model in rats: dose-dependant effect on hippocampal volume and neuron number |
| 408 | 21939841 | Electrical stimulation for epilepsy: experimental approaches |
| 409 | 8330586 | West syndrome due to perinatal insults |
| 410 | 29804730 | Perinatal insults and neurodevelopmental disorders may impact Huntington's disease age of diagnosis |
| 411 | 19333408 | Visual hallucinations: differential diagnosis and treatment |
| 412 | 24251565 | Models in research of pharmacoresistant epilepsy: present and future in development of antiepileptic drugs |
| 413 | 20618423 | In search of models of pharmacoresistant epilepsy |
| 414 | 2924747 | Phenytoin-induced seizures: a paradoxical effect at toxic concentrations in epileptic patients |
| 415 | 11956004 | Picrotoxin-induced generalised convulsive seizure in rat: changes in regional distribution and frequency of the power of electroencephalogram rhythms |
| 416 | 11762207 | Animal models of schizophrenia: a critical review |
| 417 | 18550176 | The pilocarpine model of temporal lobe epilepsy |
| 418 | 16954451 | Post-stroke seizure and post-stroke epilepsy |
| 419 | 23926279 | Posterior cingulate epilepsy: clinical and neurophysiological analysis |
| 420 | 8154869 | Posterior temporal epilepsy: electroclinical features |
| 421 | 10908200 | Role of primary sensorimotor cortices in generating inhibitory motor response in humans |
| 422 | 10924866 | Comparison between the QOLIE-31 and derived QOLIE-10 in a clinical trial of levetiracetam |
| 423 | 12639061 | Responsiveness of the quality of life in epilepsy inventory (QOLIE-89) in an antiepileptic drug trial |
| 424 | 30215021 | Responsive neurostimulation for epilepsy: More than stimulation |
| 425 | 12427900 | Singing seizures |
| 426 | 23959883 | Two distinct forms of functional lateralization in the human brain |
| 427 | 25012363 | How can we identify ictal and interictal abnormal activity? |
| 428 | 22957229 | Selective amygdalohippocampectomy |
| 429 | 24861272 | Routine developmental, autism, behavioral, and psychological screening in epilepsy care settings |
| 430 | 26365965 | A Special Electroencephalography Pattern Might Help in the Diagnosis of Antibody-positive Encephalitis |
| 431 | 6537832 | Speech arrest and supplementary motor area seizures |
| 432 | 32144451 | [Epilepsy in old age] |
| 433 | 29755904 | Radiation-induced Cavernous Malformation as a Late Sequelae of Stereotactic Radiosurgery for Epilepsy |
| 434 | 2804800 | Temporal intermittent rhythmic delta activity (TIRDA) in the diagnosis of complex partial epilepsy: sensitivity, specificity and predictive value |
| 435 | 21704564 | Benign temporo-parieto-occipital junction epilepsy with vestibular disturbance: an underrecognized form of epilepsy? |
| 436 | 12615636 | Occipital epilepsies: identification of specific and newly recognized syndromes |
| 437 | 15632275 | The out-of-body experience: disturbed self-processing at the temporo-parietal junction |
| 438 | 26002462 | Absence-like seizures and their pharmacological profile in tottering-6j mice |
| 439 | 26307329 | Neuromodulation in the Treatment of Epilepsy |
| 440 | 23002376 | 1.5 tesla magnetic resonance imaging scanners compared with 3.0 tesla magnetic resonance imaging scanners: systematic review of clinical effectiveness |
| 441 | 15572277 | Adenosine A1 receptors and the anticonvulsant potential of drugs effective in the model of 3-nitropropionic acid-induced seizures in mice |
| 442 | 20955719 | The 4-aminopyridine in vitro epilepsy model analyzed with a perforated multi-electrode array |
| 443 | 24013377 | Experimental models of status epilepticus and neuronal injury for evaluation of therapeutic interventions |
| 444 | 20951004 | Comparative anticonvulsant efficacy in the corneal kindled mouse model of partial epilepsy: Correlation with other seizure and epilepsy models |
| 445 | 2395534 | The C57BL/10Bg sps/sps mouse: a mutant with absence-like seizures; neurochemical and behavioral correlates |
| 446 | 31961886 | Health-related quality of life and its determinants among ambulatory patients with epilepsy at Ambo General Hospital, Ethiopia: Using WHOQOL-BREF |
| 447 | 23862049 | Palpitations caused by a Seizure with Autonomic Features |
| 448 | 25988019 | Ictal bradycardia and atrioventricular block: a cardiac manifestation of epilepsy |
| 449 | 22050551 | Seizure-induced miosis |
| 450 | 16060951 | Seizures manifesting as an urge to defecate, with an ictal discharge in the right hemisphere |
| 451 | 10932282 | Ictal urinary urge indicates seizure onset in the nondominant temporal lobe |
| 452 | 29686574 | Urinary urge seizure semiology localization by intracranial monitoring |
| 453 | 22957231 | Déjà experiences in temporal lobe epilepsy |
| 454 | 21478188 | Neural correlates of cognitive impairment in posterior cortical atrophy |
| 455 | 14667072 | Acquired epileptic dysgraphia: a longitudinal study |
| 456 | 11039971 | Partial seizures presenting as panic attacks |
| 457 | 15562304 | Distinguishing Lateral Temporal Neocortical and Mesial Temporal Lobe Epilepsy |
| 458 | 24967532 | Ontogenic profile of seizures evoked by the beta-carboline DMCM (methyl-6,7-dimethoxy-4-ethyl-β-carboline-3-carboxylate) in rats |
| 459 | 27657542 | The Natural History of Epilepsy in 163 Untreated Patients: Looking for "Oligoepilepsy" |

**Supplementary Table 1**: Research articles and reviews considered to extract the main concepts

- In addition, we used the following websites to enhance the ontology with more significant concepts:

| **Website name** | **URL** |
| --- | --- |
| Stanford Health Care | https://stanfordhealthcare.org/medical-conditions/brain-and-nerves/epilepsy.html |
| American Association of Neurological Surgeons | https://www.aans.org/Patients/Neurosurgical-Conditions-and-Treatments/Epilepsy |

**Supplementary Table 2**: Websites considered to collect concepts

## **Supplementary text 2: Construction of the ontology**

The EPIO Ontology was assembled using the Protégé ontology editor. This ontology is constructed based on guidelines and principles defined by Open Biological and Biomedical Ontology (OBO, http://www.obofoundry.org/) Foundry as well as aligned with the Basic Formal Ontology (BFO) hierarchy. Wherever possible, the hierarchy was based on the parent ontology from which the concept was imported. We applied Ontofox ([http://ontofox.hegroup.org](http://ontofox.hegroup.org/)) to reuse previously existing classes from other relevant ontologies. For each concept, most of the OBO ontology sources were identified, and the concepts were imported with all available annotations via Ontofox. Undefined terms in other ontologies were added with proper definitions as well as with clear provenance. In order to increase recall in Text Mining applications, we added synonyms for each concept.

**Supplementary text 3: Metadata information using annotation properties**

We added a large amount of metadata information using various annotation properties. Annotation properties were added to each entity in the ontology. These properties explain the name of the entity, standard ontology from which the entity is imported, definition of the term, source from which the term is adopted, and references that connect the term to Epilepsy. The details of the annotation properties are listed below:

- **label**: Label annotation is the unique display name of the concept.
- **oboInowl:hasDefinition**: Definitions for the concept are added under this annotation property
- **rdfs:isDefinedBy**: This annotation is added as an internal annotation to definitions, to point to the source of the definition when the definition is added manually. For modified definitions, the source of the definition is given under oboInOwl:hasDbXRef or rdfs:seeAlso.
- **oboInOwl:hasDbXRef**: To add an epilepsy specific reference link from Pubmed/NCBI
- **rdfs:seeAlso**: Any additional relevant reference links such as web articles are given under rdfs:seeAlso.
- **oboInOwl:hasExactSynonym**: Synonyms are added under this annotation property. Synonyms are terms derived from articles or research papers
- **oboInOwl:hasRelatedSynonym**: The terms that are related synonyms of the entity are added under this annotation property.

Moreover, in regards to the aforementioned annotation properties, we added some custom annotation properties to define additional information as well as to aid in applying the ontology for the use cases defined in the manuscript. Some custom annotations were used to note the source from which the terms are taken, specifying if the definition is modified from the source article of the definition and giving an explanation of the BFO hierarchy of the term. These custom annotations are listed below:

- **fromPubMed** - concepts are taken from PubMed article
- **fromArticle** - concepts are taken from resources other than PubMed
- **fromNCBIBook** - concepts are taken from NCBI Books
- **fromILAE -** concepts are taken from International League Against Epilepsy
- **fromESSO** - concept are taken from the existing ontology ESSO ([Epilepsy Syndrome Seizure Ontology](https://bioportal.bioontology.org/ontologies/ESSO))
- **fromEpSO -** concept are taken from the existing ontology EpSO ([Epilepsy and Seizure Ontology](https://bioportal.bioontology.org/ontologies/EPSO))
- **fromEPILONT -** concept are taken from the existing ontology EPILONT ([Epilepsy Ontology](https://bioportal.bioontology.org/ontologies/EPILONT))
- **CommentonDefinition** - to mention that given definition taken from the source is modified.

In addition, here is the list of annotations added to the concepts, which are used further to apply in text-mining use cases:

- **isAnatomicalEntityFor**: for concepts assigned to the “anatomical entity TM_BIN” BIN
- **isCellularProcessFor**: for concepts assigned to the “cellular process TM_BIN” BIN
- **isDiagnosisFor**: for concepts assigned to “diagnosis TM_BIN”
- **isClassificationFor:** for concepts assigned to the “epilepsy classification TM_BIN” BIN
- **isImitatorFor:** for concepts assigned to the “epilepsy imitator TM_BIN” BIN
- **isSyndromeFor:** for concepts assigned to the “epilepsy syndrome TM_BIN” BIN
- **isEtiologyFor:** for concepts assigned to the “etiology TM_BIN” BIN**.**
- **isRiksFactorFor**: for concepts assigned to the “risk factor TM_BIN” BIN
- **isseizureClassificationFor**: for concepts assigned to the “seizure classification TM_BIN” BIN
- **isSignandSymptomFor**: for concepts assigned to the “sign and symptom TM_BIN” BIN
- **isTreatmentFor**: for concepts assigned to the “treatment TM_BIN” BIN.

**Supplementary text 4: Hierarchical structure of the Epilepsy Ontology (EPIO)**

**EPIO** is a hybrid of multiple OBO Foundry ontology terms and new Epilepsy specific terms. In general, Basic Formal Ontology (BFO) (<http://basic-formal-ontology.org/>)^1^ acts as the top level ontology. Terms from existing Open Biological and Biomedical Ontology (OBO) ontologies such as OBI, OGMS, HP, and GO were integrated by the Ontofox tool^2^ in accordance to the OBO Foundry^3^ principles and the minimum information to reference an external ontology term (MIREOT) principles^4^, whenever possible (Supplementary Figure S1).

EPIO specific terms are often the terminal entries within an ontology hierarchy, comprising the leaves or parents with a short distance to the leaves. Meanwhile, OBO ontologies terms are slightly more central or internal, bridging BFO and EPIO, but they could also be terminal entries (e.g., GO related concepts).

Text Mining related BINS are located in a sidechain outside of the BFO hierarchy. Those concepts enable a collection of search terms for Text Mining applications from different branches. The concepts are related to the Text Mining BINs via specific axioms (relations).


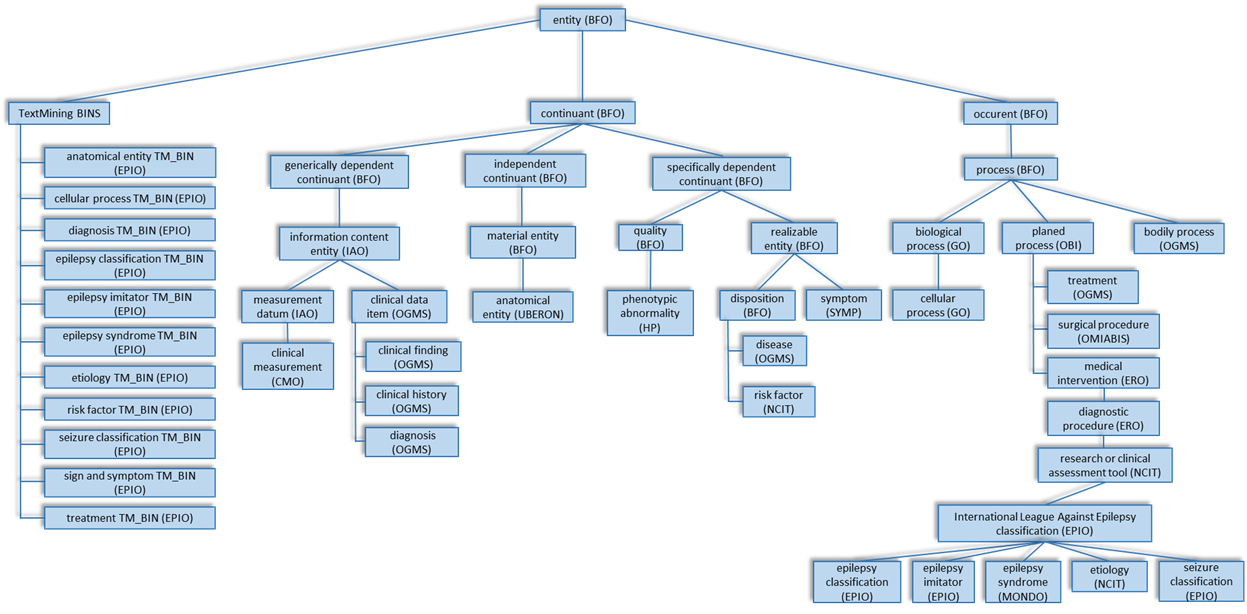


**Supplementary Figure 1**: Top hierarchical structure of EPIO.

##### **Supplementary text 5: Resources Used to Build The Ontology**

###### **International League Against Epilepsy (ILAE)**

In the first stage, all the concepts and definitions provided by International League Against Epilepsy (ILAE) (<https://www.epilepsydiagnosis.org/>) were used to build the initial version of the EPIO Ontology. ILAE provides a wide knowledge of epilepsy diagnoses and clinical aspects such as epilepsy classification, seizure classification, epilepsy syndromes, epilepsy etiologies and epilepsy imitators, as well as definitions for most of the entities listed on their website. The complete ILAE resource on epilepsy was included in the epilepsy ontology, including its maintained hierarchical structure. In total, 285 entities were imported from ILAE resources. However, there are certain clinical aspects that are not covered in ILAE resources such as screening tests, anatomy, treatment, risk factors, and symptoms. These aspects were collected from other sources.

###### **Epilepsy related ontologies**

Next, concepts from available epilepsy-related ontologies like Epilepsy and Seizure Ontology (<https://bioportal.bioontology.org/ontologies/EPSO>), ESSO (<https://bioportal.bioontology.org/ontologies/ESSO>), EPILONT (<https://bioportal.bioontology.org>/ontologies/EPILONT) were collected. Overall 350 entities from ESSO, 248 from EPSO, 33 from EPILONT were used to extend the initial ILAE-based version described above.

Epilepsy ontology (EPIO) emphasizes the reuse of terms from pre-existing OBO ontologies. Since none of these above mentioned ontologies are in the OBO foundry library (http://obofoundry.org/), priority was given to mapping the concepts to the ones present in any OBO ontologies. Altogether, 1432 entities were imported from existing OBO ontologies and 447 newly defined entities were added to Epilepsy ontology which are not present in any OBO ontology. For each entry, appropriate Epilepsy specific reference links were added.

###### **Publications and web-resources**

Additionally, epilepsy-related knowledge was manually searched in PubMed and specific concepts were added from relevant publications. A list of PMIDs from which concepts were extracted can be found in the table above (520 PubMed articles listed). The number of unique entities imported from different PubMed research articles was 459. 217 concepts were curated from research articles that are not listed in PubMed.

##### **Supplementary text 6:** **Annotation properties of concepts**

A unique primary label (rdfs:label) was added to each concept as a basic annotation, in at least one definition being added to all entities in the Epilepsy ontology. Definitions were always added using the annotation property - oboInOwl:hasDefinition. In accordance with OBO principles, entities and definitions were reused wherever possible. Definitions from ILAE were added to entities when applicable. In case a definition was present in existing ontology and also defined by ILAE, both definitions from both sources were imported to the ontology. Sources of definitions imported from sources other than an existing ontology were sub-annotated with rdfs:isDefinedBy. Database references were added with the annotation oboInOwl:hasDbXRef. Each entity was enhanced with synonyms if those were available.

**References**

Arp, R., Smith, B., & Spear, A., Building Ontologies with Basic Formal Ontology. (MIT Press, 2015).

Xiang Z, Courtot M, Brinkman RR, Ruttenberg A, He Y. OntoFox: web-based support for ontology reuse. *BMC Research Notes*. 2010, 3:175.

Smith B, Ashburner M, Rosse C, Bard J, Bug W, Ceusters W, et al. The OBO Foundry: coordinated evolution of ontologies to support biomedical data integration. Nat Biotechnol. 2007;25(11):1251–5.

Courtot M, Gibson F, Lister AL, Malone J, Schober D, Brinkman RR, et al. MIREOT: The minimum information to reference an external ontology term. *Applied Ontology*. 2011;6(1):23–33.
